# Evaluation of the Interactions between Human Serum Albumin (HSA) and Non-Steroidal Anti-Inflammatory (NSAIDs) Drugs by Multiwavelength Molecular Fluorescence, Structural and Computational Analysis

**DOI:** 10.3390/ph14030214

**Published:** 2021-03-04

**Authors:** Susana Amézqueta, José Luís Beltrán, Anna Maria Bolioli, Lluís Campos-Vicens, Francisco Javier Luque, Clara Ràfols

**Affiliations:** 1 Department of Chemical Engineering and Analytical Chemistry, Faculty of Chemistry, University of Barcelona, Martí i Franquès 1-11, 08028 Barcelona, Spain; jlbeltran@ub.edu (J.L.B.); annamariaboliolire@gmail.com (A.M.B.); crafols@ub.edu (C.R.); 2 Institute of Biomedicine (IBUB), University of Barcelona, 08028 Barcelona, Spain; fjluque@ub.edu; 3 Department of Nutrition, Food Science and Gastronomy, Faculty of Pharmacy and Food Science, University of Barcelona, Prat de la Riba 171, 08921 Santa Coloma de Gramenet, Spain; lluis.campos@pharmacelera.com; 4 Pharmacelera, Torre R, 4a planta, Despatx A05, Parc Científic de Barcelona, Baldiri Reixac 8, 08028 Barcelona, Spain; 5 Institut of Theoretical and Computational Chemistry (IQTCUB), University of Barcelona, 08028 Barcelona, Spain

**Keywords:** non-steroidal anti-inflammatory drugs, drug-protein interactions, human serum albumin, fluorescence multiwavelength data treatment, fluorescence quenching, molecular modeling

## Abstract

The interaction between drugs and transport proteins, such as albumins, is a key factor in drug bioavailability. One of the techniques commonly used for the evaluation of the drug-protein complex formation is fluorescence. This work studies the interaction of human serum albumin (HSA) with four non-steroidal anti-inflammatory drugs (NSAIDs)—ibuprofen, flurbiprofen, naproxen, and diflunisal—by monitoring the fluorescence quenching when the drug-albumin complex is formed. Two approaches—the double logarithm Stern-Volmer equation and the STAR program—are used to evaluate the binding parameters. The results are analyzed considering the binding properties, determined by using other complementary techniques and the available structural information of albumin complexes with NSAID-related compounds. Finally, this combined analysis has been synergistically used to interpret the binding of flurbiprofen to HSA.

## 1. Introduction

Drug development is a long-term process that includes five steps (discovery and development, pre-clinical research, clinical studies, regulatory approval and post-marketing safety monitoring) before a pharmaceutical product is released to the market. Once a promising compound is identified, it is necessary to gather information on its ADMET properties (absorption, distribution, metabolism, excretion, and toxicity), potential benefits, therapeutic dose, etc. [1]. Drug distribution in the bloodstream towards the target is mainly produced by reversible binding to proteins that act as carriers. The serum albumin is the most abundant protein in the blood and exerts a major role in assisting the transport of numerous endogenous compounds, such as fatty acids or hormones among body compartments, serving as a drug reservoir that can enhance drug distribution and bioavailability [2]. The drug-protein binding must be strong enough to enable drug transport, but weak enough to enable drug release to the specific target, as the free (unbound) drug fraction is the only one able to exert a therapeutic action.

Albumin is a negatively charged and water-soluble macromolecule that contains three structurally similar α-helical domains, denoted I–III, which can be further divided into subdomains A and B. Human serum albumin (HSA) has several binding sites, although drugs bind mainly to two of them: Sudlow I, also known as site 1, placed on subdomain IIA; and Sudlow II, also named as site 2, placed on subdomain IIIA. Site 1 contains a warfarin–azapropazone binding area, consisting of two overlapping binding sites for these two compounds. The non-overlapping part of the warfarin site contains a lone tryptophan residue, Trp214. Ligands strongly bound to site 1 are generally believed to be dicarboxylic acids and/or bulky heterocyclic molecules with a negative charge localized in the middle of the molecule, such as warfarin or indomethacin. On the other hand, site 2 is a largely apolar cavity with a single dominant polar patch near the pocket entrance, centered on Tyr411 and Arg410. Typical site 2 drugs are aromatic carboxylic acids with a negatively charged acid group at one end of the molecule, which is bound to a central hydrophobic moiety, such as some non-steroidal anti-inflammatory drugs (NSAIDs; ibuprofen, diflunisal, flurbiprofen, naproxen, etc.) [3]. Moreover, it is important to note that drugs can bind with different affinity to several pockets of the protein [4,5], and that there can exist competition for sites during the coadministration of drugs [3,6,7]. Even HSA pockets can be occupied by other undesirable exogenous compounds, such as some mycotoxins that could interfere with the drug transport [8].

Drug-albumin interactions can be evaluated using several complementary techniques, such as isothermal titration calorimetry (ITC), fluorescence spectroscopy (FS), capillary electrophoresis/frontal analysis (CE-FA), or equilibrium dialysis (ED) [9]. In the case of FS, albumin is considered to be the main fluorophore, as it contains three fluorescent amino acids: Tryptophan, tyrosine, and phenylalanine, the two first present in the main binding sites. The Stern–Volmer (SV) or the double logarithm Stern–Volmer (DLSV) equations can be used to relate the fluorescence quenching event with the binding parameters (binding constant and stoichiometry). These equations present several drawbacks, as they consider albumin as the sole fluorophore, assuming that only one type of interaction is formed, and that the concentration of the free drug is much higher than the bound fraction. However, there often exist other fluorophores in solution (such as the drug or a drug-albumin complex) that make it necessary to work under more selective, but less sensitive conditions [10]. To overcome these drawbacks, other strategies should be used. As an example, the binding constants can also be evaluated by considering the whole fluorescence spectrum (i.e., taking into account the contribution of all the possible species in equilibrium), as exemplified by the STAR program [11,12].

This work determines the binding parameters of some drug-HSA complexes measured by FS using both strategies (DLSV and the STAR program) and compare their applicability in the absence or presence of spectral interferences owed to drug fluorescence. The results are discussed in light of the binding affinities determined by using other techniques, such as isothermal calorimetry (ITC) and capillary electrophoresis frontal analysis (CE/FA), as well as other fluorescence-based binding data reported in previous studies. Finally, the results are examined in light of the available structural information about albumin complexes with NSAID and NSAID-related compounds, in conjunction with molecular docking.

## 2. Results and Discussion

### 2.1. Setup of Experimental Conditions

**Figure 1:**
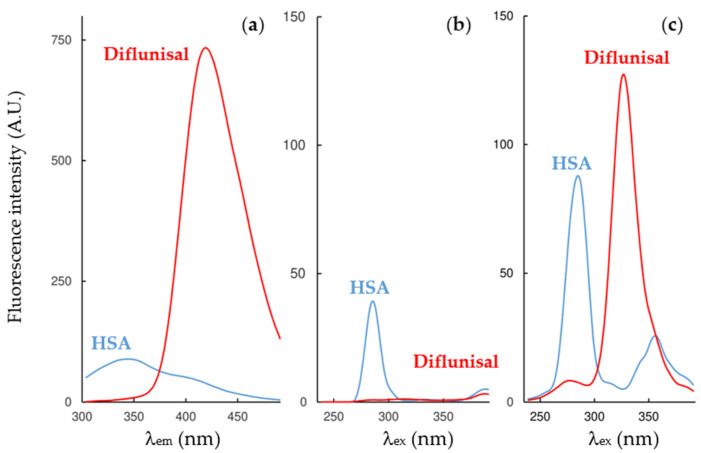
Comparison of fluorescence spectra in the study of diflunisal-HSA: (**a**) Emission; (**b**) synchronous Δ*λ* 15 nm; (**c**) synchronous Δ*λ* 60 nm modes at 25 °C.

To select the optimal wavelengths for the DLSV approach, the single spectrum of each compound has been evaluated. HSA shows maximum fluorescence in the emission mode at 346 nm (*λ*_ex_ = 285 nm), and in the synchronous modes at 285–286 nm. Table 1 shows the percentage of interferences (calculated as *I*_drug_/*I*_total_ × 100) produced by the inner fluorescence of the drugs over the HSA at the wavelength of its maximum fluorescence intensity. On the one hand, flurbiprofen shows significant interferences at the three modes of work, and naproxen in the emission mode and in the Δ*λ* 60 nm synchronous mode. On the other, ibuprofen and diflunisal show minor interferences at the three modes, and naproxen in the Δ*λ* 5 nm synchronous mode. As an example, the fluorescence spectra of diflunisal and HSA at 25 °C at the three fluorescence modes of work are shown in Figure 1.

The wavelengths selected for the DLSV approach and the corresponding spectral differences are indicated in Table 1. The criterion for selection is to choose the wavelength with the maximum protein fluorescence signal and the minimum drug fluorescence interference. We assume that the interferences under 10% can be evaluated using the DLSV equation. In the case of ibuprofen and diflunisal (at the three modes), and naproxen (synchronous ∆*λ* = 15 nm), it is possible to work at wavelengths near the maximum fluorescence intensity of the albumin. In the case of naproxen using the emission mode, it is possible to use the DLSV approach in conditions not far from the albumin fluorescence maximum, with the drawback to having lower sensitivities, and hence, higher experimental errors. Last, as flurbiprofen shows significant spectral interferences with the albumin in the whole range of work, it is not possible to evaluate this drug using the DLSV approach. The interactions of naproxen using the synchronous mode at ∆*λ* = 60 nm cannot be evaluated either with this approach.

### 2.2. Fluorescence Measurements for Drug-Albumin Interaction

**Figure 2:**
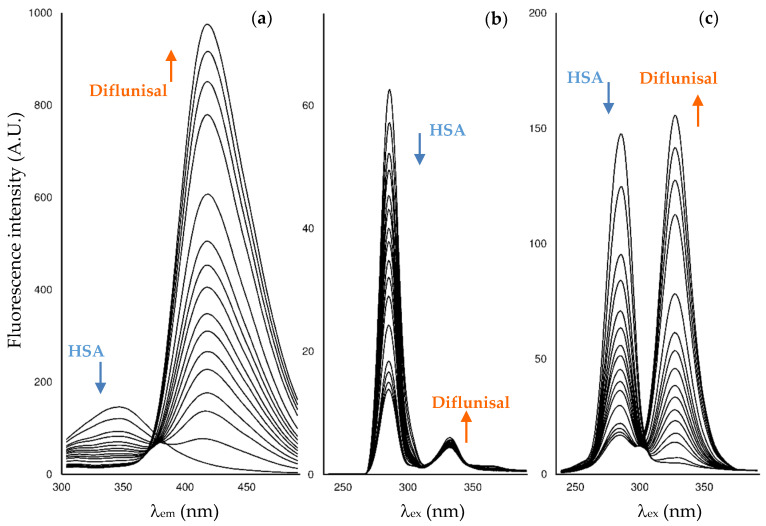
Fluorescence spectrum for diflunisal-HSA at: (**a**) Emission; (**b**) synchronous Δ*λ* = 15 nm; (**c**) synchronous Δ*λ* = 60 nm modes. The sixteen additions of diflunisal performed to cover the ratio 0.7:13 with respect to the initial concentration of HSA in the cell (4.5 μM).

When successive amounts of the studied drugs are added to a fixed concentration of HSA, the albumin fluorescence is quenched. As an example, Figure 2 shows the changes in the fluorescence spectra for HSA after the addition of increasing amounts of diflunisal. Here, HSA experiences fluorescence decay when the quencher is added, and the drug-protein complex is formed. On its behalf, as diflunisal also has fluorophore groups that emit in the emission and synchronous Δ*λ* 60 nm modes, the fluorescence of the free-drug form increases when an excess of diflunisal is added to the cuvette (the fluorescence study on the other two NSAIDs is shown in Supporting Information Figure S1). 

The binding parameters have been calculated by evaluating the quenching event using first the DLSV approach (25 °C). In a first instance, we have checked that the formation of the ground-state complex occurs, as the bimolecular quenching constant obtained from the SV equation is higher than the maximum value possible for diffusion-limited quenching in water (∼10^10^ M^−1^ s^−1^) for all the HSA-drug experiments (data not shown). Afterwards, the interactions have been evaluated by the DLSV equation (Equation (3)), considering the data obtained from the different fluorescence modes separately (Table 2).

Next, the interactions have been quantified using the STAR program approach (at 20, 25, and 37 °C) (Table 3). Here, due to the data handling ability of the program, the data from the different fluorescence modes has been treated simultaneously to obtain more precise binding parameters values. The simultaneous evaluation has been possible because in an initial data treatment, we did not observe significant differences in the binding results between the three fluorescence modes for the NSAIDs under study. 

Ibuprofen. Fluorescence data, treated whether with the DLSV approach or the STAR program, have not provided quantitative binding data values for the HSA-ibuprofen complex. In the first case, a log *K*_b1_ in the order of 2–3 units could be intuited, although the error associated with the calculation was of the same order of magnitude. In the second, the errors during the mathematical evaluation of the data were high and did not detect any binding event (Figure S1.1 in Supporting Information shows the small changes in the spectra produced with the addition of ibuprofen). Other complementary techniques, such as equilibrium dialysis, capillary electrophoresis, and ITC ([13] and Table 4 and Table 5), indicate that the HSA-ibuprofen interaction occurs, and the associated binding constant is of the same order of magnitude as the binding constant determined for other NSAIDs. The reason for this difference is that the fluorescence quenching technique only evaluates those interactions that occur in the environment of the fluorophores. Therefore, it can be concluded that those interactions reported in the literature take place far from the fluorophores, particularly Trp214, present in the binding sites of HSA.

Naproxen. The STAR program approach has shown log *K*_b_ values of ~4.8. Although previous studies show that naproxen can bind to albumin at different binding sites [13], the best model obtained with the STAR program contains only one interaction. Moreover, the analysis of experimental data of the fluorescence quenching study treated with the STAR program at different temperatures shows that the binding constant is not temperature-dependent in the working range (20–37 °C). With respect to the DLSV approach, the slightly lower values obtained by this second method (log *K*_b_ ~ 4.0) could be due to the restriction of the model to deal with spectral interferences coming from the drug itself. 

As suggested in the literature, the fluorescence quenching studies have been performed at different acquisition modes to putatively distinguish between the contributions of the different fluorophores (emission for the global fluorescence, synchronous at Δ*λ* = 15 nm for Tyr and synchronous at Δ*λ* = 60 nm for Trp). Using either DLSV or STAR program approaches, the binding constant values do not vary to a high extent considering the three modes of acquisition. 

In a previous study performed by recording the fluorescence decay by using time-correlated single-photon counting (TCSPC), the log *K*_b_ value obtained for the HSA-naproxen complex was 5.59 [16]. This log *K*_b_ value is slightly lower than the main binding event obtained by ITC by our research group (5.95; Table 4). The second binding event quantified by ITC (log *K*_b2_ = 4.85) and by CE-FA is similar to the value obtained by FS in the present study using the STAR program approach, and slightly higher than the one obtained by the DLSV approach.

Diflunisal. The results using the STAR program show the presence of two interactions with an affinity difference of one order of magnitude (log *K*_b1_ ~ 5.7 and log *K*_b2_ ~ 4.7, respectively). The log *K*_b1_ value agrees with those reported in the literature (Table 5 and [19]), although there are differences in the stoichiometry. When evaluating the data using the DLSV approach, only one interaction with an average value between log *K*_b1_ and log *K*_b2_ obtained with the STAR program is observed (log *K*_b_ ~ 5.2). This value is similar to the one obtained when limiting the equilibrium model in STAR to a single possible interaction (data not shown) and to the one reported in the literature using the DLSV data treatment approach [17]. Consequently, when multiple species are associated with the binding process, the DLSV approach calculates the main binding event, whereas the STAR program studies all the species in equilibrium. As the other NSAIDs considered in the present work, the binding values do not show temperature dependence in the working range, or a variation using different FS acquisition modes.

Flurbiprofen. In the case of HSA-flurbiprofen binding, the DLSV approach could not be used to perform the data treatment, due to the drug-related interferences (Table 1). The STAR program can deal with the data with one restriction: The 1:1 binding equilibria had to be calculated considering the emission and synchronous Δ*λ* = 15 nm mode and fixed, and next, the 1:2 binding equilibria was calculated using the data of the three fluorescence modes. The reason is that the synchronous Δ*λ* = 60 nm mode offered spectra with such high intensities that they could not be considered together with those of the two other modes in a first instance. 

Results show that there exist two binding events (1:1 and 1:2, respectively) with close binding constant values (both in the order of log *K*_b_ ~ 5). These values are very similar at the three temperatures considered, and hence, it is not possible to evaluate thermodynamic parameters using the van’t Hoff equation. The binding values agree with other values reported in the literature [13], though there are differences in the stoichiometry. If we consider the values that we obtained previously (Table 4), the *K*_b_ determined by FS corresponds to the *K*_b2_ value obtained by ITC and CE-FA. We assume that the first interaction obtained by ITC corresponds to the interaction that occurs in the main binding site of flurbiprofen, in an attachment point free from fluorophores in the nearby. As other binding events with different stoichiometries and binding constant values have been observed with different complementary techniques (Table 4 and [13]), there would exist other binding sites with a lower affinity to flurbiprofen.

### 2.3. Structural Analysis of NSAID-HSA Complexes

**Figure 3:**
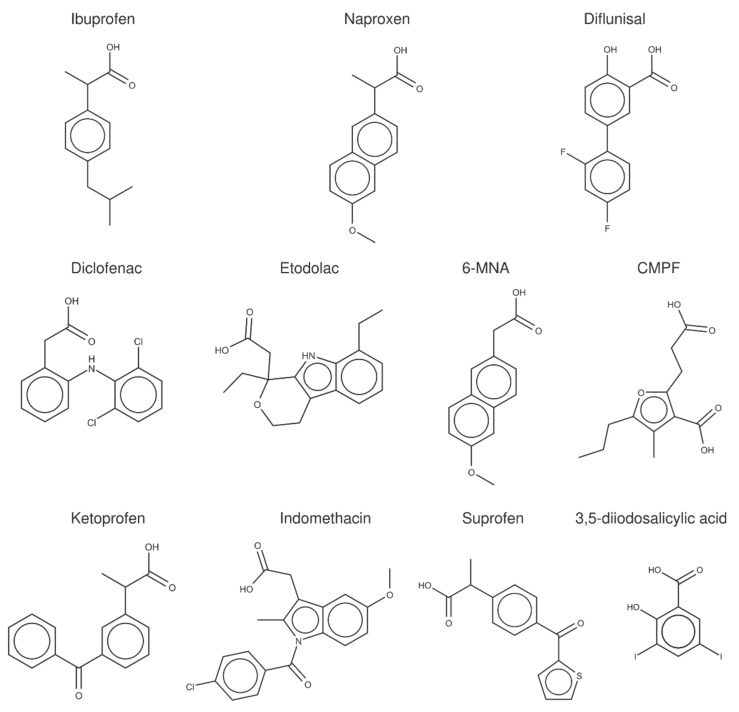
Chemical structures of the NSAIDs and related compounds included in the analysis of X-ray crystallographic structures.

To examine the data obtained from FS measurements and the complementary techniques reported in the literature (Table 4 and Table 5), a crystallographic analysis of the albumin complexes with (*S*)-ibuprofen, (*S*)-naproxen, diflunisal, and a set of structurally related compounds (Figure 3) was performed.

**Figure 4:**
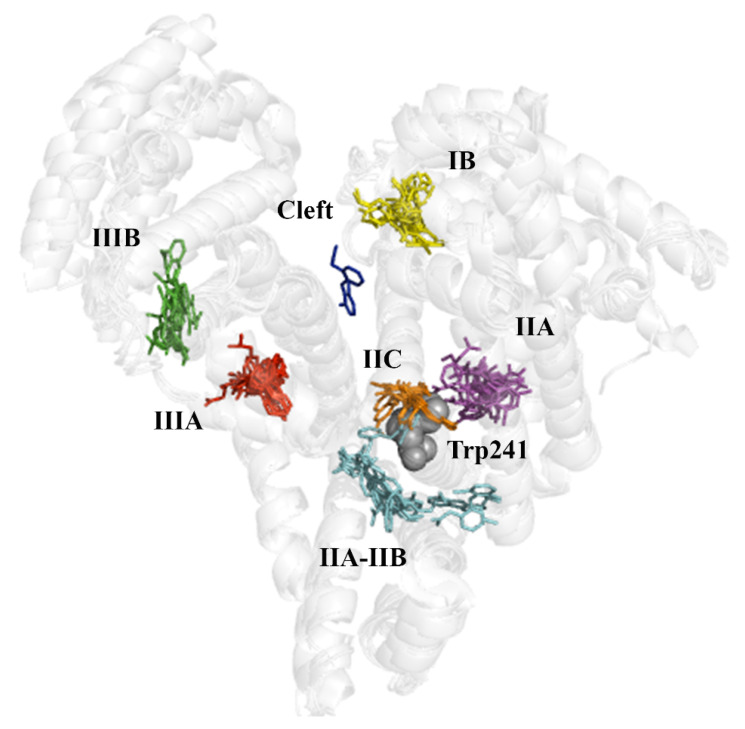
Representation of the main clusters observed for (*S*)-ibuprofen, (*S*)-naproxen, diflunisal, and NSAID-related compounds in the X-ray crystallographic structures of their complexes with albumin from different organisms. The protein backbone of selected albumins (PDB entries 2BXG, 6OCI, 4OT2, 4LUH, 6OCL, and 5OSW; see also Table 6) are shown as a light gray cartoon. The only tryptophan residue (Trp241) present in human serum albumin is shown as gray spheres. Ligands bound to a common binding site are shown in different colors.

In the last years, previous studies have examined the binding of fatty acids, endogenous compounds, and a variety of drugs to albumin [3,20], expanding the previous work by Ghuman et al. [5]. For our purposes here, the X-ray crystallographic analysis was specifically focused on compounds that exhibit an amphiphilic character, sharing a negatively charged carboxylate group bound directly or through a short methylenic chain to an aromatic ring. Furthermore, with the exception of 3,5-diiodosalicylic acid, they have a similar size and shape, as noted in an average surface and volume of 306 ± 44 Å^2^ and 229 ± 33 Å^3^ (Supporting Information Table S1). On the other hand, the X-ray crystallographic structures correspond to the complexes formed with HSA (8 structures), but also with *Equus caballus* (ESA; 13), *Bos taurus* (BSA; 4), *Ovis aries* (2), *Capra Hircus* (1) and *Oryctolagus cuniculus* (2), leading to 31 X-ray complexes (Table 6). The inclusion of these complexes was motivated by the preservation of the global fold of the protein backbone (see Figure 4), the high sequence identity found between the albumins of these organisms with HSA, which is higher than 75% (>87% when conservative changes are considered), and particularly to the preservation of the residues that shape the binding pockets (Supporting Information Table S2).

The superposition of all the X-ray crystallographic complexes reveals that the ligands are grouped in seven distinct clusters, which are shown in Figure 4 following the numbering reported by Ghuman et al. [5]. One of them is the cleft found between domains I and III, and corresponds to the binding of diclofenac to the ovine serum albumin (PDB entry 6HN0). In contrast, the other binding pockets exhibit a variety of bound ligands, supporting the ability of serum albumin to accommodate structurally related chemical scaffolds of the series of compounds, shown in Figure 3, including the occupancy of several pockets by the same compound, as has been noticed for other molecules [21].

Ibuprofen. In HSA (PDB entry 2BXG), (*S*)-ibuprofen is bound to two binding sites, denoted as sites IIIA and IIA-IIB (see Supporting Information Figure S2). In IIIA, the carboxylate group forms electrostatic interactions with Arg410 (distance of 2.8 Å from the guanidinium unit) and Lys414 (distance of 3.0 Å from the amino group), respectively, and forms a direct hydrogen bond (distance of 2.7 Å) with the hydroxyl group of Tyr411. In ESA (PDB entries 6OCI and 6U4X), the arrangement of the (2-methylpropyl)benzene unit is different in 6OCI and 6U4X, due to a conformational change of Tyr410 (equivalent to Tyr411 in HSA), although the interactions formed by the carboxylate moiety, especially regarding the hydrogen bond formed with Tyr410 (2.9 Å), are retained. On the other hand, the binding mode of (*S*)-ibuprofen at site IIA-IIB is more variable. Thus, in HSA, the carboxylate group forms a salt bridge with Lys351 and a hydrogen bond with the backbone NH unit of Val482, but in ESA, the ligand adopts a different arrangement, as the carboxylate group is stabilized by a salt bridge with Arg208. The closest distance from the indole ring of Trp214 to the center of the benzene ring in (*S*)-ibuprofen is 10.6 and 18.9 Å for IIA-IIB and IIIA, respectively. 

Naproxen. Inspection of the X-ray structures of the complexes of (*S*)-naproxen bound to ESA (PDB entries 4OT2, 4ZBR, and 5DBY) shows that the ligand binds to pockets in sites IIIA and IIA-IIB (see Supporting Information Figure S3). In ESA-IIIA, the carboxylate group forms electrostatic interactions with Arg409 and Lys413, with distances of 3.3 and 3.8 Å from the guanidinium and amino groups, respectively, supplemented by a hydrogen bond with the hydroxyl group of Tyr410. This binding mode is also observed in the X-ray structure of the complex of naproxen with BSA (PDB entry 4OR0). Binding of (*S*)-naproxen to site IIA-IIB is observed in X-ray structures 4OT2 and 4ZBR, but not in 5DBY, suggesting a weaker binding to this pocket. In site IIA-IIB, the carboxylate group interacts with Lys350 (distance of 3.6 and 4.1 Å in 4OT2 and 4ZBR), and forms hydrogen bonds with the hydroxyl group of Ser479 and the backbone NH units of Leu480 or Ala481. Finally, binding of (*S*)-naproxen to sites IIIA and IIA-IIB is impeded by the presence of molecules of decanoic acid that occupy these pockets in HSA (PDB entry 2VDB). Instead, (*S*)-naproxen fills the pocket IB, with the carboxylate group close to the guanidinium moiety of Arg186 (at 4.2 Å), although this binding mode may be affected by the presence of a decanoic acid molecule in the same pocket.

Interestingly, the X-ray structure of the BSA complexed with (*S*)-naproxen reveals the occurrence of an additional binding site located between subdomains IIA-IIB and IIA (named IIC in Figure 4), where the naphthalene ring of the ligand is partially stacked against the indole ring of Trp213 (equivalent to Trp214 in HSA), the shortest distance being ~3.6 Å, and the carboxylate group is stabilized by electrostatic interactions with Arg194, Arg198, and Ag217, and hydrogen bonded to the indole NH unit (distance of 2.8 Å).

Diflunisal. This ligand binds three pockets in HSA (PDB entry 2BXE): IIIA, IIA-IIB, and IIA (see Supporting Information Figure S4). In site IIIA, the carboxylate group is stabilized by salt bridges with Arg410 and Lys414 (distances of 3.0 and 3.3 Å), and the hydroxyl group of the ligand forming a hydrogen bond (2.5 Å) with the hydroxyl group of Tyr411. In site IIA-IIB, binding is assisted by a double hydrogen bond of the carboxylate group with the backbone NH units of Leu481 and Val482, which supplements the electrostatic interaction with Lys351 (distance of 3.6 Å). Finally, in site IIA, the carboxylate group is hydrogen bonded to Arg257 (2.7 Å), His242 (3.7 Å), and Tyr150 (2.2 Å; note that this short distance may be affected by the lower accuracy of the electron density around the carboxylate moiety of the ligand). Here the shortest distance from the center of the difluorobenzene ring of diflunisal to the indole ring of Trp214 is 6.8 Å.

### 2.4. Evaluation of the Ligand Binding Events for NSAID-HSA Complexes

Ibuprofen. Since ibuprofen does not significantly modify the albumin fluorescence, the structural data suggest that binding to HSA takes place far from the main fluorophore (Trp214, shown as gray spheres in Figure 4) present in HSA. Thus, the closest distance of (*S*)-ibuprofen from the indole ring of Trp214 is 10.6 Å upon binding to site IIA-IIB, which increases to almost 19 Å when bound to site IIIA. According to these distances, one may expect a low efficiency in the quenching of Trp214 fluorescence, especially for the binding to site IIIA, since the efficiency of fluorescence emission quenching generally requires distances between Trp and quencher lower than 10 Å [22]. Moreover, the interaction at site IIA-IIB can be expected to be weak, since different binding modes are observed in PDB entries 2BXG (HSA) and 6OCI (ESA), which cannot be attributed to species-related differences, since the residues that define the walls of site IIA-IIB in HSA and ESA are identical. Rather, they likely arise from the small size of this drug and the presence of several anchoring points in this pocket. On the other hand, although a Tyr residue is present in site IIIA, the quenching effect would not be enough to get quantitative binding data by fluorescence. If fluorescence quenching occurs in these two sites, it is hardly detectable, due to the distance to the fluorophore and the weak binding to a specific point in the protein. These putative weak interactions would be consistent with the ibuprofen-HSA binding values obtained using CE/FA [13]. In this case, a weak *K*_b_ value with a stoichiometry of *n* = 5 was found, which would prove the ability of ibuprofen to bind to several sites with roughly equivalent affinity. In addition, it would justify why very weak interactions near a fluorophore are only intuited by fluorescence quenching in the present study.

Naproxen. The single interaction observed by fluorescence quenching, which is characterized by a log *K_b_* value of 4.80 (Table 3), can be ascribed to the interaction event that occurs in site IIA-IIB. According to the X-ray data in PDB entries 4OT2, 4ZBR, and 4OR0, the closest distance between the aromatic ring of (*S*)-naproxen and the main fluorophore (Trp213 in ESA and BSA, equivalent to Trp214 in HSA) ranges from 8.4 to 9.6 Å. Therefore, the stronger binding observed by ITC (log *K_b_* of 5.95; Table 4) can be ascribed to site IIIA, where the distance between the aromatic rings of (*S*)-naproxen and Trp213 is close to 17 Å. On the other hand, although the literature suggests that naproxen could diffuse into site IIA [16], we have not observed this interaction upon inspection of the available crystallographic data. The additional interactions detected by ITC (*n* = 2.5) and CE/FA (*n* = 3.5) [13] may reflect similar weak interactions at other sites that are quantified at once using calorimetry or electrophoretic measurements. As indicated in the literature, naproxen would have only one high-affinity binding site (IIIA) and several low-affinity binding sites [23]. In the case of the secondary binding events, binding of the ligand might involve different conformations depending on the experimental conditions [23].

As a final remark, it is worth noting that the binding to site IIC observed in the complex with BSA (PDB entry 4OR0) might justify the quenching of the Trp214 phosphorescence described in previous studies [23]. In particular, the quenching of Trp214 phosphorescence has been interpreted resorting to a Dexter energy transfer mechanism, which requires orbital overlap of fluorophore and quencher, and hence, a short distance between (*S*)-naproxen and Trp214, which would agree with the close contact observed between the aromatic rings of (*S*)-naproxen and Trp214 in the X-ray data.

Diflunisal. The fluorescence quenching technique identifies two interactions with HSA characterized by log *K_b_* values of 5.86 and 4.70, which could be attributed to the binding to sites close to Trp214. As noted above, one of them would be site IIA, as suggested by the X-ray structure 2BXE, which reveals a short ring-to-ring distance from the ligand to Trp214 (around 6.8 Å). We propose that the other binding site could be IIA-IIB, as the shortest distance from the indole ring of Trp214 to the aromatic ring of diflunisal is 8.7 Å. 

**Figure 5:**
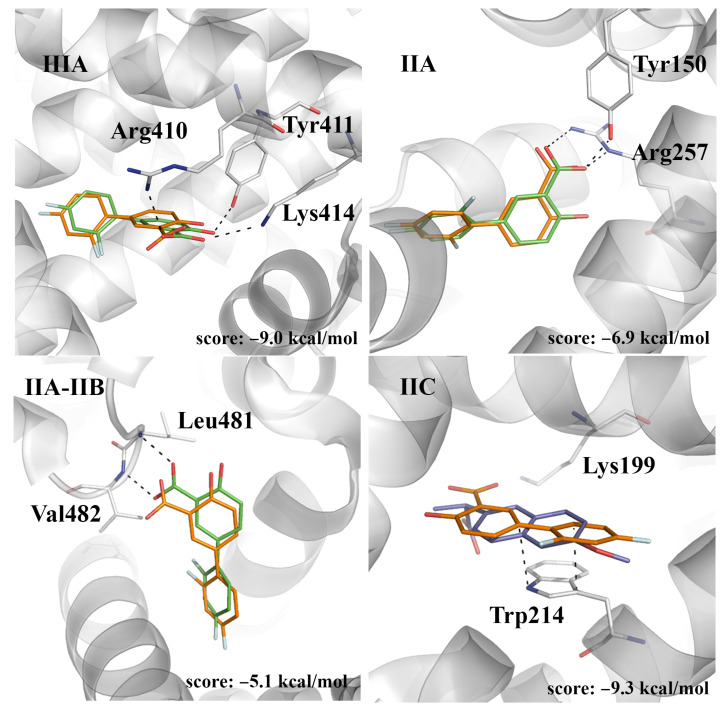
The predicted pose of diflunisal bound to sites IIIA, IIA, IIA-IIB, and IIC. The crystallographic poses of diflunisal (bound at sites IIIA, IIA, and IIA-IIB; PDB entry 2BXE), and naproxen (bound at site IIC; PDB entry 4OR0) are shown as sticks (C atoms in green and dark blue, respectively). The docked pose of diflunisal is shown with C atoms as orange sticks. The protein backbone is displayed as a gray cartoon. Selected interactions with HSA residues are represented as dashed lines.

Although there is no crystallographic evidence showing the binding of diflunisal to site IIC, docking calculations support the binding to this pocket with a docking score of –9.3 kcal/mol (Figure 5). The predicted arrangement of diflunisal superposes well the structure of (*S*)-naproxen bound to site IIC in BSA, as noted in the overlay of the aromatic rings present in diflunisal and (*S*)-naproxen, revealing that the difluorobenzene moiety of diflunisal stacks against the indole ring (average distance of 3.7 Å). Furthermore, the similar arrangement of the carboxylate moiety in these compounds could be stabilized by electrostatic interactions with Lys195 and Lys199. Albeit caution is needed for quantitative comparison, due to the known deficiencies of the docking scoring functions, let us note that this pose has a better score than the crystallographic poses at sites IIA (–6.9 kcal/mol) and IIA-IIB (–5.1 kcal/mol), whereas it compares with the score of the pose at site IIIA (–9.0 kcal/mol). This may suggest that binding to site IIC may be kinetically slow, requiring dynamical rearrangements of certain structural elements of the protein. Accordingly, studies focused on the quenching of the Trp214 phosphorescence would be valuable to elucidate the potential binding of diflunisal to site IIC.

Finally, let us remark that other authors already noticed the multiplicity of binding events of diflunisal to HSA by using potentiometric measurements with *K*_b_ values similar to the ones found by our group of research [19]. Indeed, they reported an occupancy of *n* = 4.28 for the strongest binding event that may correspond to the union of diflunisal to different sites with similar affinity.

### 2.5. Prospective Study of the Flurbiprofen-HSA Complex

On the basis of the preceding analysis, we have revisited the fluorescence results observed for the flurbiprofen-HSA complex, which revealed the occurrence of two binding events with similar binding affinities (both in the order of log *K*_b_ ~ 5; see Table 4). In particular, the *K*_b_ values obtained by FS are in agreement with the *K*_b2_ attained by ITC (Table 4), and the stronger binding affinity obtained by ITC (log *K*_b1_ of 6.70) should involve a site distant from fluorophores in HSA, particularly Trp241.

**Figure 6:**
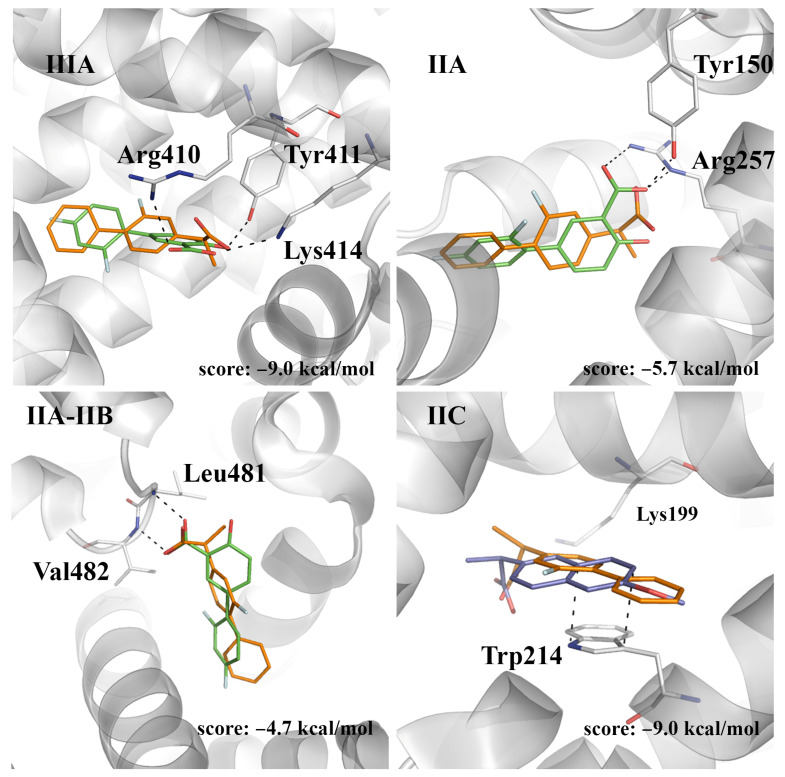
The predicted pose of (*S*)-flurbiprofen bound to sites IIIA, IIA, IIA-IIB, and IIC. The crystallographic poses of diflunisal (bound at sites IIIA, IIA, and IIA-IIB; PDB entry 2BXE), and (*S*)-naproxen (bound at site IIC; PDB entry 4OR0) are shown as sticks (C atoms in green and dark blue, respectively). The docked pose of (*S*)-flurbiprofen is shown with C atoms as orange sticks. The protein backbone is displayed as a gray cartoon. Selected interactions with HSA residues are represented as dashed lines.

Docking computations reveal that (*S*)-flurbiprofen binds to site IIIA matching the arrangement observed for (*S*)-naproxen and (*S*)-ibuprofen in this cavity, enabling the formation of a hydrogen bond between the carboxylate group of (*S*)-flurbiprofen and the hydroxyl group of Tyr411 (Figure 6; score of −9.0 kcal/mol). According to the results previously discussed for the other compounds, we assume that this interaction should have little quenching effect on the fluorescence of HSA, due to the large distance from Trp214. On the other hand, docking in IIA and IIC leads to poses with scores of −5.7 and −9.0 kcal/mol, respectively. It is worth noting that (*S*)-flurbiprofen exhibits a large overlap with the X-ray crystallographic pose of (*S*)-naproxen at site IIC, and diflunisal at site IIA (Figure 6). Finally, docking of (*S*)-flurbiprofen in site IIA-IIB leads to a binding mode that superposes well with diflunisal (score of −4.7 kcal/mol; Figure 6).

**Figure 7:**
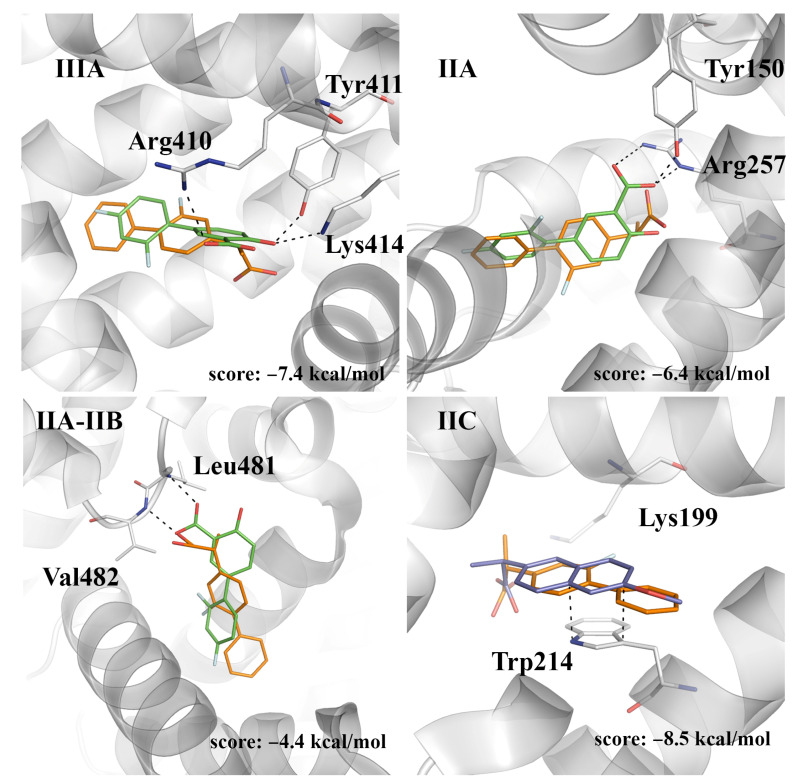
The predicted pose of (*R*)-flurbiprofen bound to sites IIIA, IIA, IIA-IIB, and IIC. The crystallographic poses of diflunisal (bound at sites IIIA, IIA, and IIA-IIB; PDB entry 2BXE) and (*S*)-naproxen (bound at site IIC; PDB entry 4OR0) are shown as sticks (C atoms in green and dark blue, respectively). The docked pose of (*R*)-flurbiprofen is shown with C atoms as orange sticks. The protein backbone is displayed as a gray cartoon. Selected interactions with HSA residues are represented as dashed lines.

With regard to (*R*)-flurbiprofen, site IIC appears to be the best binding site, leading to a close overlap of both the carboxylate moieties and aromatic rings of both (*R*)-flurbiprofen and (*S*)-naproxen (Figure 7). The score of this pose (−8.5 kcal/mol) compares with the value obtained for (*S*)-flurbiprofen in this pocket (−9.0 kcal/mol), whereas binding to site IIIA leads to a reduced score (−7.4 kcal/mol), which can be understood from the weakening of electrostatic interactions between the carboxylate unit and both Lys414 and Arg410. Lower scores are obtained for the binding to sites IIA and IIA-IIB.

Taking into account the analysis reported above for ibuprofen, naproxen, and diflunisal, present results suggest that the strongest binding site observed from ITC measurements (log *K*_b_ ~ 6.7) may primarily correspond to the binding of flurbiprofen to site IIIA, where the shortest distance from the ligand to Trp214 is 19 Å, which would lead to a weak quenching efficiency. The two binding signals determined with fluorescence assays for the racemic sample of flurbiprofen (log *K*_b_ ~ 5.5) may arise from the interaction of the two enantiomers at sites IIA or IIA-IIB, as the shortest distance from Trp214 range from 6 to 9 Å. Previous studies had already indicated that there exist spectral differences upon the interaction of (*S*)- and (*R*)-flurbiprofen, with HSA, which can be interpreted from distinctive signatures in the binding of the two enantiomers [16,24,25]. At this point, it is worth noting that a recent theoretical study provided evidence that supports the ability of the two enantiomeric species to adopt similar binding modes at site IIA-IIB, and that this interaction justifies the quenching of Trp214 fluorescence, whereas the enantioselective quenching efficiency would arise from the increased flexibility of (*S*)-flurbiprofen in the binding pocket [26].

As noted above, docking calculations suggest a favorable binding to site IIC. Occupancy of this pocket, however, is scarcely observed in the X-ray crystallographic data. This may obey to factors, such as the occurrence of dicarboxylic acids, which may block access to this site, as found in the X-ray structure 4OT2, which corresponds to the complex of ESA with (*S*)-naproxen bound to sites IIIA and IIA-IIB (Supporting Information Figure S5). Similar cosolutes are also found at the entrance of site IIC in other structures, such as 4ZBQ, 4ZBR, 5DBY, and 6OCI (data not shown). Furthermore, the presence of other molecules bound to albumin may trigger structural changes that alter the shape of pocket IIC, as found in PDB entries 2VDB, 4Z69, and 2BXL, due to the binding of several molecules of palmitic and pentadecanoic acids to HSA (Supporting Information Figure S6).

**Figure 8:**
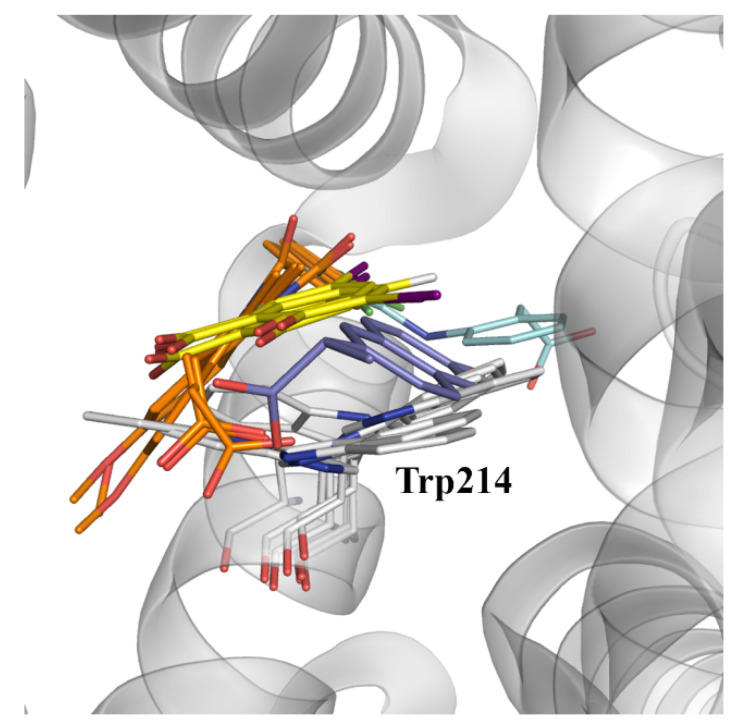
Representation of the binding mode of (*S*)-naproxen, indomethacin, 3,5-diiodosalicylic acid, and diclofenac (these ligands are shown as sticks with C atoms in dark blue, orange, yellow, and cyan, respectively) to binding site IIC in albumin (taken from X-ray structures 4OR0, 2BXK, 2BXM, 2BXQ, 4JK4, 4LUH, 5OSW, and 4Z69). The orientation of the side chain of Trp214, which is found in two distinct conformations, is highlighted as sticks. The protein backbone is displayed as a gray cartoon.

In spite of these considerations, a detailed inspection of the available X-ray structures reveals the ability of pocket IIC to accommodate a variety of ligands (Figure 8), such as indomethacin (PDB entries 2BXK, 2BXM, and 2BXQ), 3,5-diiodosalycilic acid (PDB entries 4JK4, 4LUH, and 5OSW), diclofenac (PDB entry 4Z69), in addition to the complex with (*S*)-naproxen (PDB entry 4OR0).

Let us note that the diversity of binding modes found for these compounds may be related to the plasticity of this pocket, as noted in the different orientations adopted for the side chain of Trp214, which can adopt two distinct conformations related by a rotation of 180 degrees around the Cβ-Cγ bond (Figure 8). Therefore, it may be speculated that binding to site IIC may require a dynamical adjustment of the protein, leading to a kinetically slower binding that presumably may involve a preliminary binding to site IIA and the subsequent migration to site IIC, or alternatively the selective binding to distinct conformational states of the protein [27,28,29].

Whereas, the larger solvent exposure of sites IIIA and IIA-IIB would facilitate a faster recognition and binding of NSAID-related compounds, enabling the effective quenching of Trp214 fluorescence upon binding to site IIA-IIB, binding to site IIC would provide an explanation to the effective quenching of Trp214 phosphorescence observed for (*S*)-naproxen and flurbiprofen, according to the requirement of orbital overlap between donor and acceptor in the Dexter mechanism of electron transfer [16,24,25].

## 3. Materials and Methods

### 3.1. Equipment

FS measurements were performed on a Cary Eclipse Fluorescence Spectrophotometer from Varian-Agilent Technologies (Santa Clara, CA, USA) using a 1 cm path length quartz QS cuvette (Hellma Analytics, Jena, Germany), 600 nm/min scan speed, slit widths of 5 nm for both excitation and emission monochromators, and Savitzky-Golay filter was applied to smooth the experimental data. The temperature was kept constant by using a magnetic heater stirrer (Agimatic-N, JP Selecta, Abrera, Spain).

A Crison micro-pH 2002 potentiometer (Crison Instruments, Alella, Spain) equipped with a Crison 5014 combination electrode was used for pH measurements. The electrode system was calibrated with standard buffers of pH 4.01 and 7.00.

### 3.2. Reagents

HSA (>99%), (*R*,*S*)-ibuprofen (>98%), (*S*)-naproxen (>98%), diflunisal (>98%), and (*R*,*S*)-flurbiprofen (>98%) were obtained from Sigma-Aldrich (St Louis, MO, USA). Hereafter, unless specified in the text, the stereochemical nature of the compounds will be omitted for the sake of simplicity. The purity of albumin was verified by spectrophotometry [30]. Drugs and albumin were dissolved in phosphate buffer saline (PBS) (pH 7.40, 10 mM phosphate, 140 mM of chloride, and 150 mM ionic strength) at the desired concentration. The p*H* was adjusted with 0.5 M HCl (Titrisol, Merck). Na_2_HPO_4_ (>99%) and KH_2_PO_4_, KCl, NaCl (>99.5%) were from Merck. Water purified by a Milli-Q-plus system from Millipore (Bedford, MA, USA) with a resistance higher than 18 MΩ·cm was used to prepare the solutions.

### 3.3. Fluorescence Quenching/Enhancement Measurement

The FS measurement conditions were set up taking into account that (i) the albumin fluorescence study must be performed in a linear concentration-fluorescence range, (ii) the availability of HSA is limited, (iii) the drug must be soluble in the working conditions, and (iv) the drug may contain fluorophore groups and interfere in the albumin fluorescence measurement. In the working conditions, albumin had a linear fluorescence-concentration behavior from 0.5 to 8 μM (data not shown). Therefore, the quenching/enhancement was evaluated using intermediate concentration levels (3 or 5 μM, depending on the experiment). The drugs considered showed a limited solubility in PBS, and therefore, the stock solutions were prepared at 1000 μM for the most soluble drugs (ibuprofen, naproxen, and diflunisal), and at 300 μM for the least soluble one (flurbiprofen). These protein and drug concentration levels measure the specific binding of the drug to the protein and do not correspond to the plasmatic ones. In fact, the usual albumin concentration in plasma is about 600 μM, and the drug:albumin ratio at the therapeutic drug dose is usually as low as 1:100 for ibuprofen, 1:250 for flurbiprofen, 1:500 for naproxen [31], and 1:30 for diflunisal [32].

To perform each experiment, an albumin solution aliquot (2700 μL) was poured into the cuvette that was placed in a water bath over a magnetic heater stirrer. Fluorescence quenching/enhancement data were obtained as emission spectra (*λ*_ex_ = 285 nm, *λ*_em_ = 295–550 nm), and synchronous scanning with Δ*λ* = 15 nm (*λ*_em_= 230–400 nm), and Δ*λ* = 60 nm (*λ*_em_ = 230–400 nm) to gather information of the whole molecule, the Tyr environment, and the Trp environment, respectively [33], and to reduce the drug interferences in the later albumin spectra. To use the double logarithmic Stern–Volmer equation, single excitation-emission wavelength pairs were used. For the STAR program, the whole spectrum was considered.

Next, sixteen consecutive additions of the corresponding drug solution were made (the solution concentration and the added volumes varied depending on the drug evaluated) to cover a drug:albumin concentration ratio in the range 0.4–14. This ratio covered the maximum stoichiometry expected, 9.7 μM [13]. Moreover, drug concentrations had to be limited to avoid the signal saturation of the fluorescence detector caused by very fluorescent drugs and to enable the data treatment of the whole spectrum. Fluorescence quenching experiments did not reach a plateau, a constant fluorescence signal, but this fact did not avoid data treatment using whether the DLSV or the STAR program approaches. The cuvette was maintained in the water bath for 150 s after the addition to reach the equilibrium and let the temperature stabilize. The cuvette was quickly placed into the fluorimeter, and the fluorescence spectra were measured at the conditions indicated previously. Experiments were carried out in duplicate under constant stirring (400 rpm), and at three different temperatures (20, 25, and 37 °C), which were chosen to encompass both the standard range of room temperature and the temperature in the human body.

### 3.4. Fluorescence Quenching/Enhancement Measurement

In the present study, we have evaluated the drug-protein interaction using two different approaches based on fluorescence quenching measurements (we did not observe fluorescence enhancement).

Fluorescence quenching refers to any process that reduces the fluorescence intensity of a sample. A variety of molecular interactions, including excited-state reactions, molecular rearrangements, energy transfer, ground-state complex formation, and collisional quenching, can result in fluorescence quenching [34].

The quenching effect can be described with the SV Equation (1)

(1) $$\frac{F_{0}-F}{F}=K_{q}\times \;\tau \times \left[Q\right]$$

where *F*_0_ and *F* are the fluorescence intensity in the absence and the presence of a quencher, respectively; *K*_q_ is the bimolecular quenching constant (M^−1^ s^−1^); τ is the average lifetime of the molecule without the quencher (its value is in the range 2–7 ns [35]); and [*Q*] is the quencher (drug) concentration (M).

Ground-state complex formation is produced when the bimolecular quenching constant obtained from the SV equation is higher than the maximum value possible for diffusion-limited quenching in water (∼10^10^ M^−1^ s^−1^) [36]. In the present work, the bimolecular quenching constant is over the diffusion-limited quenching in water, and therefore, the drug-protein binding event occurs and can be evaluated.

#### 3.4.1. Double Logarithm Stern–Volmer Equation

The double logarithm SV Equation (2) is a linearization of the Hill equation.

(2) $$\log\frac{F_{0}-F}{F}=\log K_{b}+n_{H}\log\left[Q_{f}\right]$$

where *K*_b_ is the binding constant; *n*_H_ is the Hill coefficient; and [*Q*_f_] is the free quencher (drug) concentration.

Usually, the free drug concentration [*Q*_f_] is unknown, and it is replaced by the total drug concentration [*Q*] (i.e., assuming that the concentration of the macromolecule is negligible with respect to the drug concentration; Equation (3).

(3) $$\log\frac{F_{0}-F}{F}=\log K_{b}+n_{H}\log\left[Q\right]$$

This equation only yields the binding stoichiometry in the case of “infinite” cooperativity. It is well known that this equation can be used to fit the data even if the hypothesis of “infinite” cooperativity is not satisfied, but in this case, *n*_H_ is just a phenomenological parameter, which is lower than the real number of binding sites [37]. Further, it considers that the only fluorophore in solution is the protein and dismisses the possible contribution of the drug or the drug-protein complex to the fluorescence. Therefore, the applicability of the DLSV equation is restricted to these scenarios.

In the present work, the Hill coefficients and the binding constants were obtained from the log ((*F*_0_ − *F*)/*F*) vs. log [*Q*] plot by using MS Excel^TM^ (Microsoft, Redmond, WA, USA) worksheet.

#### 3.4.2. STAR Program

The extended version of the non-linear least squares program STAR [11] can handle spectral data from multiple equilibria systems containing up to 300 solutions, measured at 500 data points. In this case, an equilibrium system can be described by a set of components (as albumin, quencher, hydrogen ion, etc.) that can form different species. These species are defined by their stoichiometric coefficients, the corresponding binding constants, and their spectral characteristics Equation (4).

(4) $$sS+qQ\;↔\;S_{s}Q_{q}\;K_{b}=\frac{\left[S_{s}Q_{q}\right]}{{\left[S\right]}^{s}{\left[Q\right]}^{q}}$$

Moreover, the experimental data for each solution contains the total concentrations of components and the measured fluorescence spectrum.

On the other hand, the fluorescence spectrum of a given solution is equal to the sum of the fluorescence spectra of all species. If we have a set of *n* fluorescent species, STAR solves the mass balances of the components, calculating the concentrations for all species for the given set of binding constants. Then, the fluorescence intensity at a *j* wavelength can be calculated as indicated in Equation (5):

(5) $$If_{j}=\overset{n}{\underset{i=1}{\sum }}C_{i}\cdot \Phi_{i,j}$$

where *C_i_* indicates the concentration of the i-species, and $\Phi_{i,j}$ is the molar fluorescence of the i-species at the j-wavelength. The $\Phi_{i,j}$ values can be supplied as data input, or calculated by the program.

The binding constants are refined using the Gauss–Newton iterative algorithm until a minimum of the sum of squared differences (*U* in Equation (6)) between the experimental and calculated fluorescence intensities is obtained:

(6) $$U=\overset{ns}{\underset{i=1}{\sum }}\overset{nw}{\underset{j=1}{\sum }}{\left(If_{i,j,\exp}-If_{i,j,\mathrm{calc}}\right)}^{2}$$

where *ns* and *nw* indicate the number of solutions and wavelengths, respectively; the subscripts exp and calc indicate the experimental values and those values calculated by the program, respectively.

The program output includes the standard deviation of the errors between experimental and calculated data, together with the estimated error in the binding constants.

The extended version of the STAR program allows the data treatment of the full fluorescence spectra at the three modes of work simultaneously, calculating the binding constants and the unitary spectrum of the species formed. In this way, the drawbacks owed to the application of the DLSV equation can be overcome.

In the present work, the stoichiometry and the binding constant of the drug-protein complexes have been calculated using the STAR program considering the whole spectral data (including the emission spectrum, and the two synchronous fluorescence spectra with Δ*λ* = 15 nm and Δ*λ* = 60 nm, respectively).

#### 3.4.3. Docking Calculations

Docking calculations were performed to examine the binding of selected compounds (diflunisal, flurbiprofen) to HSA using the 2019-2 release of Glide [38,39]. The crystal structure of HSA was retrieved from the Protein Data Bank (PDB code: 2BXE [5]). This structure contains three molecules of diflunisal bound to binding sites IIA, IIA-IIB, and IIIA. The protein structure was prepared by adding bond orders and adding hydrogen atoms, followed by restrained energy minimization, using the Protein Preparation Wizard module in Maestro [40]. The ligands were modeled as the anionic species using LigPrep [41]. The binding sites were enclosed in grids defined with an inner box of 10 Å × 10 Å × 10 Å, and the extra precision (XP) GlideScore was used to evaluate the quality of the poses [42]. Default settings were used for all remaining parameters. The results of docking simulations were visually examined using PyMOL software [43].

## 4. Conclusions

In the present work, the interactions between four NSAIDs and their main transporter in the human blood, the human serum albumin, have been evaluated. The study has been performed by evaluating the fluorescence quenching caused by the formation of the drug-protein complex using two different approaches (the DLSV equation and the STAR program). Although both approaches show similar results, the STAR program is able to overcome some restrictions of the DLSV equation. This program evaluates the interactions in the presence of spectral interferences produced by the drug or the drug-protein complexes formed (as shown in the case of naproxen or flurbiprofen), and can consider the interaction in more than one or one group of equivalent sites (as shown in the case of diflunisal). The comparison of the results with those obtained with other complementary techniques, such as ITC denotes the importance of evaluating the interactions with different complementary techniques. In the present case, fluorescence is well suited to evaluate selectively the interactions that occur near the protein fluorophores, particularly Trp214 for HSA. Furthermore, the integration of the information derived from these complementary techniques is valuable to shed light on the complexity of the binding events that mediate the interaction of compounds with HSA, which may be affected by the variety of pockets present in serum albumin and the conformational flexibility of this protein. In this sense, the results derived from the synergic combination between different experimental and computational techniques may clarify the interpretation of accumulated ligand binding data and gain insight into the functional role played by HSA in the transport of bioactive compounds and drugs.

## Figures and Tables

**Table 1:** Percent spectral interferences produced by the different non-steroidal anti-inflammatory drugs (NSAIDs) over the human serum albumin (HSA) spectrum at the wavelengths that provide the maximum fluorescence signal and at the optimal experimental conditions.

Fluorescence Mode	Emission Wavelength	Ibuprofen	Flurbiprofen	Naproxen	Diflunisal
Emission mode, *λ*_*ex*_ = 285 nm	Maximum	346 nm (interf. 7.9%)	346 nm (interf. 53%)	346 nm (interf. 91%)	346 nm (interf. 8.2%)
	Optimum	As maximum	- ^1^	322 nm (interf. 5.3%)	As maximum
Synchronous mode, ∆*λ* = 15 nm	Maximum	286 nm (interf. 1.1%)	286 nm (interf. 85%)	286 nm (interf. 6.4%)	285 nm (interf. 2.1%)
	Optimum	As maximum	-	As maximum	As maximum
Synchronous mode, ∆*λ* = 60 nm	Maximum	286 nm (interf. 7.4%)	286 nm (interf. 52%)	286 nm (interf. 90%)	285 nm (interf. 7.9%)
	Optimum	As maximum	-	-	As maximum

^1^ Not optimum conditions—found that spectral interferences over 10% were present in the whole work range.

**Table 2:** Stoichiometry and binding constants obtained at 25 °C using the DLSV approach for data at three different fluorescence modes.

System	Emission		Synchronous Δ*λ* = 15 nm		Synchronous Δ*λ* = 60 nm	
	n_H1_	log *K*_b1_	n_H1_	log *K*_b1_	n_H1_	log *K*_b1_
HSA-Ibuprofen	NQ ^1^	NQ	NQ	NQ	NQ	NQ
HSA-Flurbiprofen	- ^2^	-	-	-	-	-
HSA-Naproxen	0.94 (0.01)	4.2 (0.1) ^3^	0.89 (0.01)	3.70 (0.08) ^3^	-	-
HSA-Diflunisal	1.01 (0.02)	5.2 (0.1) ^3^	1.09 (0.02)	5.1 (0.1) ^3^	1.04 (0.02)	5.3 (0.1) ^3^

^1^ NQ, not quantifiable; ^2^ not evaluated due to drug spectral interferences over 10%; ^3^ the determination coefficient (*R*^2^) of the regression used to evaluate this parameter is ~0.98.

**Table 3:** Stoichiometry and binding constants obtained using the STAR program approach considering the three selected fluorescence modes simultaneously. *K*_b1_ and *K*_b2_ correspond to the binding constants of 1:1 and 1:2 stoichiometry processes, respectively. Standard deviations are noted in parentheses.

System	Temperature	log *K*_b1_	log *K*_b2_
HSA-Ibuprofen	20–37 °C	ND ^1^	
HSA-Flurbiprofen ^2^	20 °C	4.91 (0.01)	5.53 (0.01)
	25 °C	4.96 (0.02)	5.52 (0.01)
	37 °C	4.98 (0.02)	5.22 (0.01)
HSA-Naproxen	20 °C	4.88 (0.01)	
	25 °C	4.80 (0.01)	
	37 °C	4.66 (0.01)	
HSA-Diflunisal	20 °C	5.74 (0.01)	4.57 (0.01)
	25 °C	5.86 (0.01)	4.70 (0.01)
	37 °C	5.62 (0.01)	4.49 (0.01)

^1^ ND, not detected; ^2^ the 1:1 binding equilibria are calculated considering the emission and synchronous Δ*λ* = 15 nm mode and fixed, next the 1:2 binding equilibria are calculated using the data of the three fluorescence modes.

**Table 4:** Stoichiometry and binding constants obtained for the NSAIDs interactions with HSA and *Bos taurus* (BSA) using isothermal titration calorimetry (ITC) and capillary electrophoresis/frontal analysis (CE-FA) (HEPES 50 mM, I = 50 mM, pH = 7.4, T = 25 °C) [13].

Drug	*n*_1_	log *K*_b1_	*n*_2_	log *K*_b2_	*n*_3_	log *K*_b3_
*Ibuprofen*						
HSA ITC	0.84	5.95	-	-	-	-
HSA CE/FA	-	-	5.2	4.9	-	-
BSA ITC	0.8	5.90	-	-	-	-
BSA CE/FA	-	-	7.2	4.2	-	-
*Naproxen*						
HSA ITC	1.00	5.95	2.5	4.85	-	-
HSA CE/FA	-	-	3.5	4.9	-	-
BSA ITC	0.81	7.17	-	-	-	-
BSA CE/FA	-	-	4.0	4.2	-	-
*Flurbiprofen*						
HSA ITC	0.71	6.70	4.8	4.78	-	-
HSA CE/FA	-	-	5.0	4.5	-	-
BSA ITC	0.80	6.3	-	-	-	-
BSA CE/FA	-	-	6.4	4.3	8.6	3.9

**Table 5:** Stoichiometry and binding constants were reported in the literature for the NSAIDs interactions with HSA using molecular fluorescence.

Drug	*n*_1_	log *K*_b1_	pH	T (°C)	Buffer (Concentration)	Ref.
Ibuprofen	1	3.92 ^1^	7.4	25	PBS (50 mM)	[14]
Ibuprofen	1.18	6.39 ^1^	7.4	25	PBS (67 mM)	[15]
Naproxen	1	5.59	7.4	NA ^2^	PBS (10 mM)	[16]
Diflunisal	1	5.16	NA	NA	NA	[17]
Diflunisal	1	5.93 ^3^	7.0	25	15 mM trisodium citrate (I = 150 mM)	[18]

^1^ data obtained by competitive binding method; ^2^ NA, Data not available; ^3^ log *K*_SV_ (Stern–Volmer constant).

**Table 6:** X-ray crystallographic structures included in the structural analysis of albumin complexed with ibuprofen, naproxen, diflunisal, and NSAID-related compounds.

PDB Code	Organism	Resolution (Å)	Ligand	Binding Sites
2BXA	*Homo sapiens*	2.35	CMPF	IIIA, IIA
2BXE	*Homo sapiens*	2.95	Diflunisal	IIIA, IIA-IIB, IIA
2BXG	*Homo sapiens*	2.70	(*S*)-Ibuprofen	IIIA, IIA-IIB
2BXK	*Homo sapiens*	2.40	Indomethacin	IIC
2BXL	*Homo sapiens*	2.60	3,5-diiodosalicylic acid	IIA
2BXM	*Homo sapiens*	2.50	Indomethacin	IB, IIC
2BXQ	*Homo sapiens*	2.60	Indomethacin	IB, IIC
2VDB	*Homo sapiens*	2.52	(*S*)-Naproxen	IB
4Z69	*Homo sapiens*	2.19	Diclofenac	IIA-IIB, IIA, IB
4J2V	*Equus caballus*	2.12	3,5-diiodosalicylic acid	IIIA, IIA, IB, IIIB
4ZBQ	*Equus caballus*	1.92	Diclofenac	IIIA, IIIB
4ZBR	*Equus caballus*	2.19	Diclofenac(*S*)-Naproxen	IIIBIIIA, IIA-IIB
4OT2	*Bos taurus*	2.42	(*S*)-Naproxen	IIIA, IIA-IIB
5DBY	*Equus caballus*	2.35	Diclofenac(*S*)-Naproxen	IIIBIIIA
5V0V	*Equus caballus*	2.45	Etodolac	IIA-IIB, IIA, IB
6OCI	*Equus caballus*	2.54	(*S*)-Ibuprofen	IIIA, IIA-IIB
6OCJ	*Equus caballus*	2.50	Suprofen	IIIA
6U4R	*Equus caballus*	2.45	Ketoprofen	IIIB
6U4X	*Equus caballus*	2.25	(*S*)-Ibuprofen	IIIA
6U5A	*Equus caballus*	2.65	6-MNA	IIIA, IIA-IIB, IIIB
4JK4	*Bos taurus*	2.65	3,5-diiodosalicylic acid	IIIA, IIA, IB, IIC
4OR0	*Bos taurus*	2.58	(*S*)-Naproxen	IIIA, IIA-IIB, IIC
6QS9	*Bos taurus*	2.80	Ketoprofen	IIA
6OCK	*Oryctolagus cuniculus*	1.90	Ketoprofen	IIIA, IIIB
6OCL	*Oryctolagus cuniculus*	2.35	Suprofen	IIIA
4LUH	*Ovis aries*	2.20	3,5-diiodosalicylic acid	IIA, IIC
6HN0	*Ovis aries*	2.12	Diclofenac	IIIA, IIA-IIB, IB, IIIB, Cleft
5OSW	*Capra haircus*	1.78	3,5-diiodosalicylic acid	IIIA, IIA, IB, IIIB, IIC

## Data Availability

Data is contained within the article and supplementary material.

## Supplementary Materials

The following are available online at https://www.mdpi.com/1424-8247/14/3/214/s1, Figure S1: Fluorescence spectrum for (1) ibuprofen-HSA, (2) naproxen-HSA and (3) flurbiprofen-HSA at 25 °C and at: (a) Emission; (b) synchronous Δ*λ* = 15 nm; (c) synchronous Δ*λ* = 60 nm modes, Figure S2: Representation of the binding mode of (*S*)-ibuprofen at binding sites IIA and IIA-IIB in HSA (PDB entry 2BXG) and ESA (PDB entries 6U4X and 6OCI), Figure S3: Representation of the binding mode of (*S*)-naproxen at binding sites IIIA, IIA-IIB, IB, and IIC in ESA (PDB entries 4OY2, 4ZBR, and 5DBY), BSA (PDB entry 4OR0) and HSA (PDB entry 2VDB), Figure S4: Representation of the binding mode of diflunisal at binding sites IIIA, IIA-IIB, and IIA in HSA (PDB entry 2BXE), Figure S5: Representation of the X-ray structure 4OT2, which corresponds to HSA with diclofenac bound at sites IIA and IIA-IIB, Figure S6: Superposition of the X-ray structures of HSA 2BXE and 4Z69, Table S1: Surface (Å2) and volume (Å3) of the compounds selected for structural analysis, Table S2: Comparison of sequence similarity between the serum albumin of different organisms compared to the human protein.
