# Supplementary material for: Evaluation of the Interactions between Human Serum Albumin (HSA) and Non-Steroidal Anti-Inflammatory (NSAIDs) Drugs by Multiwavelength Molecular Fluorescence, Structural and Computational Analysis
Source: Pharmaceuticals (Basel). 2021 Mar 4;14(3):214. doi: 10.3390/ph14030214 (PMC8000696; doi:10.3390/ph14030214)
Supplement: Supplementary file 1 [file pharmaceuticals-14-00214-s001.pdf]

**Supporting Information**

# EVALUATION OF THE INTERACTIONS BETWEEN HUMAN SERUM ALBUMIN (HSA) AND NON-STEROIDAL ANTI-INFLAMMATORY (NSAIDS) DRUGS BY MULTIWAVELENGTH MOLECULAR FLUORESCENCE, STRUCTURAL AND COMPUTATIONAL ANALYSIS

**Susana Amézqueta[a,b,*], Jose Luís Beltrán[a], Anna Maria Bolioli[a], Lluís Campos-Vicens[c,d], Francisco Javier Luque[b,c,e], Clara Ràfols[a,b]**

[a] Department of Chemical Engineering and Analytical Chemistry, Faculty of Chemistry, University of Barcelona, Martí i Franquès 1-11, 08028 Barcelona, Spain.

[b] Institute of Biomedicine (IBUB), University of Barcelona.

[c] Department of Nutrition, Food Science and Gastronomy, Faculty of Pharmacy and Food Science, University of Barcelona, Prat de la Riba 171, 08921 Santa Coloma de Gramenet, Spain

[d] Pharmacelera, Torre R, 4a planta, Despatx A05, Parc Científic de Barcelona, Baldiri Reixac 8, 08028 Barcelona, Spain.

[e] Institut of Theoretical and Computational Chemistry (IQTCUB), University of Barcelona.

**Figure S1.** Fluorescence spectrum for (1) ibuprofen-HSA, (2) naproxen-HSA and (3) flurbiprofen-HSA at 25ºC and at: (a) emission; (b) synchronous Δλ=15 nm; (c) synchronous Δλ=60 nm modes.

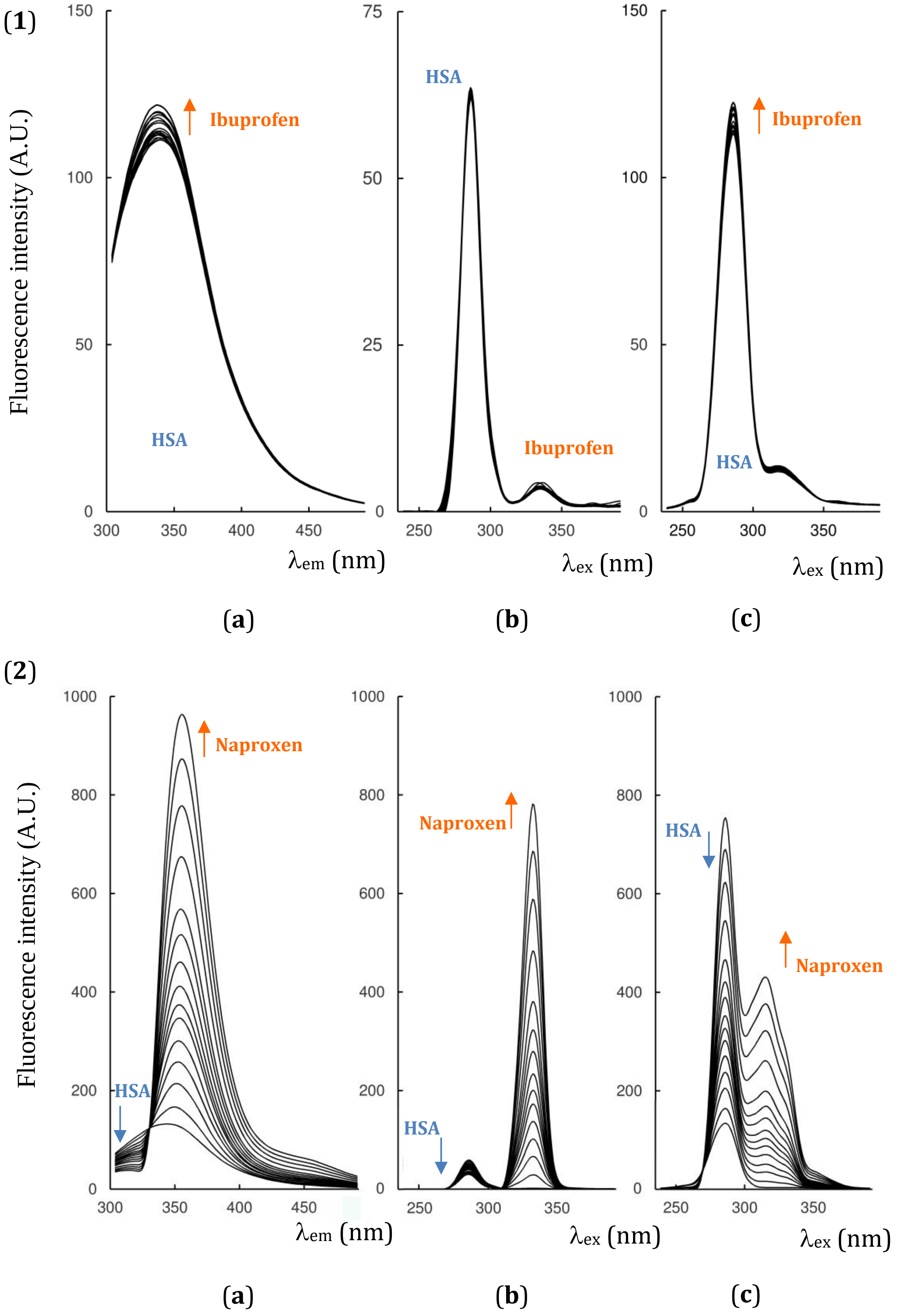

**(3)**

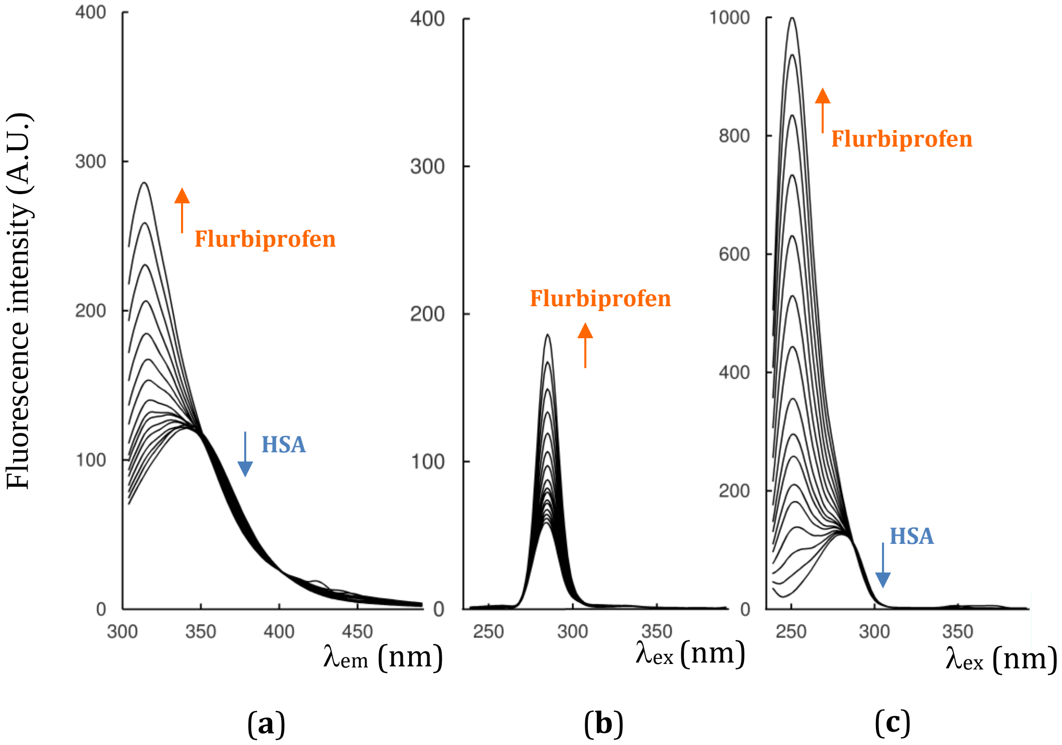
Fluorescence intensity (A.U.)
400
300
200
100
0
300
350
400
450
λem (nm)
Flurbiprofen
HSA
(a)
400
300
200
100
0
250
300
350
λex (nm)
Flurbiprofen
(b)
1000
800
600
400
200
0
250
300
350
λex (nm)
Flurbiprofen
HSA
(c)

**Figure S2.** Representation of the binding mode of (*S*)-ibuprofen at binding sites IIA and IIA-IIB in HSA (PDB entry 2BXG) and ESA (PDB entries 6U4X and 6OCI). The crystallographic pose of (*S*)-ibuprofen is shown with C atoms as colored sticks. Selected interactions with HSA residues are represented displaying the residues with C atoms as grey sticks, and interactions between residues as dashed lines. The protein backbone is displayed as grey cartoon. See Table 6 for details of binding site occupancy.

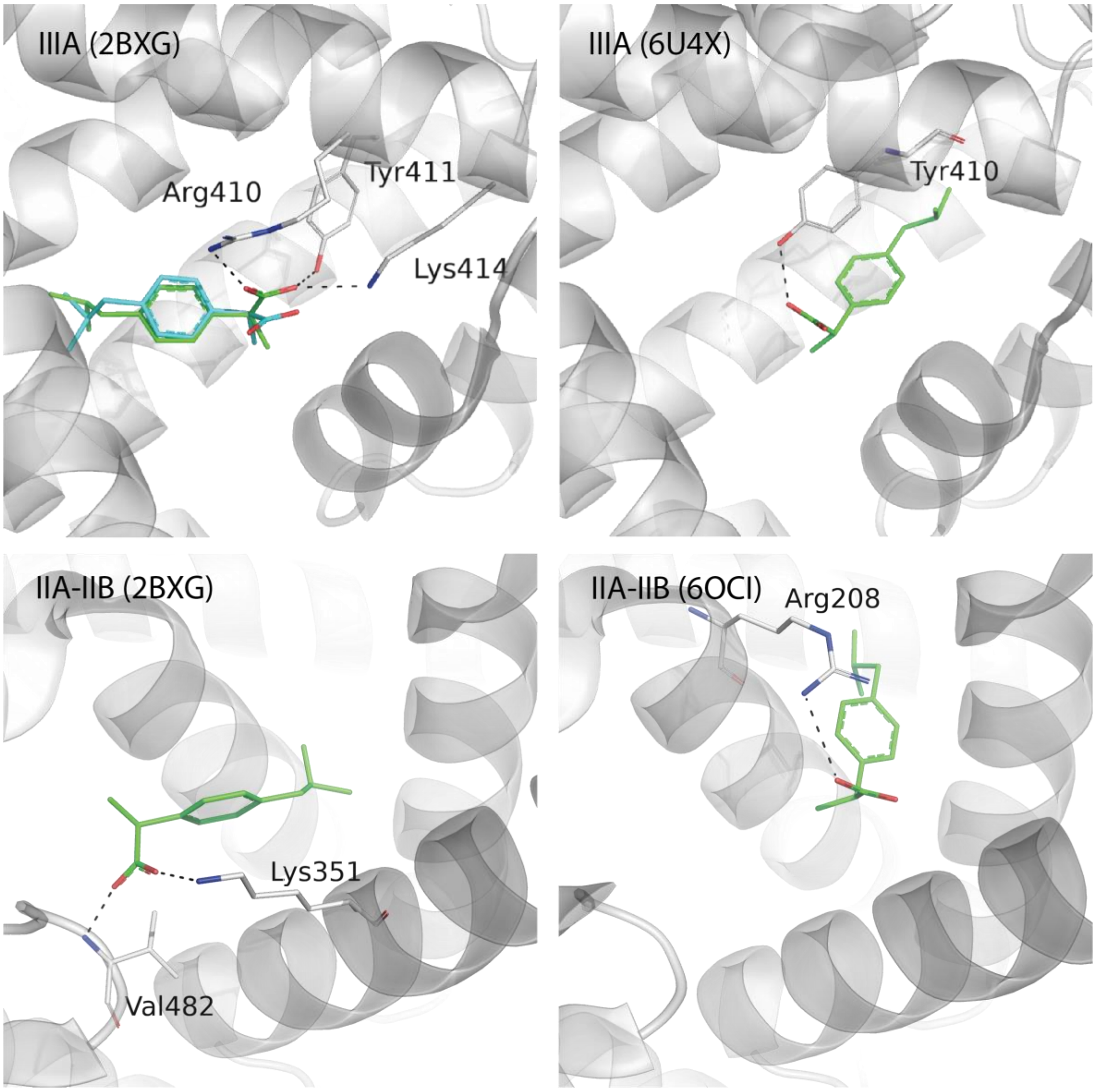

**Figure S3.** Representation of the binding mode of (*S*)-naproxen at binding sites IIIA, IIA-IIB, IB and IIC in ESA (PDB entries 4OY2, 4ZBR and 5DBY), BSA (PDB entry 4OR0) and HSA (PDB entry 2VDB). The crystallographic pose of (*S*)-naproxen is shown with C atoms as colored sticks. Selected interactions with HSA residues are represented displaying the residues with C atoms as grey sticks, and interactions between residues as dashed lines. The protein backbone is displayed as grey cartoon. See Table 6 for details of binding site occupancy.

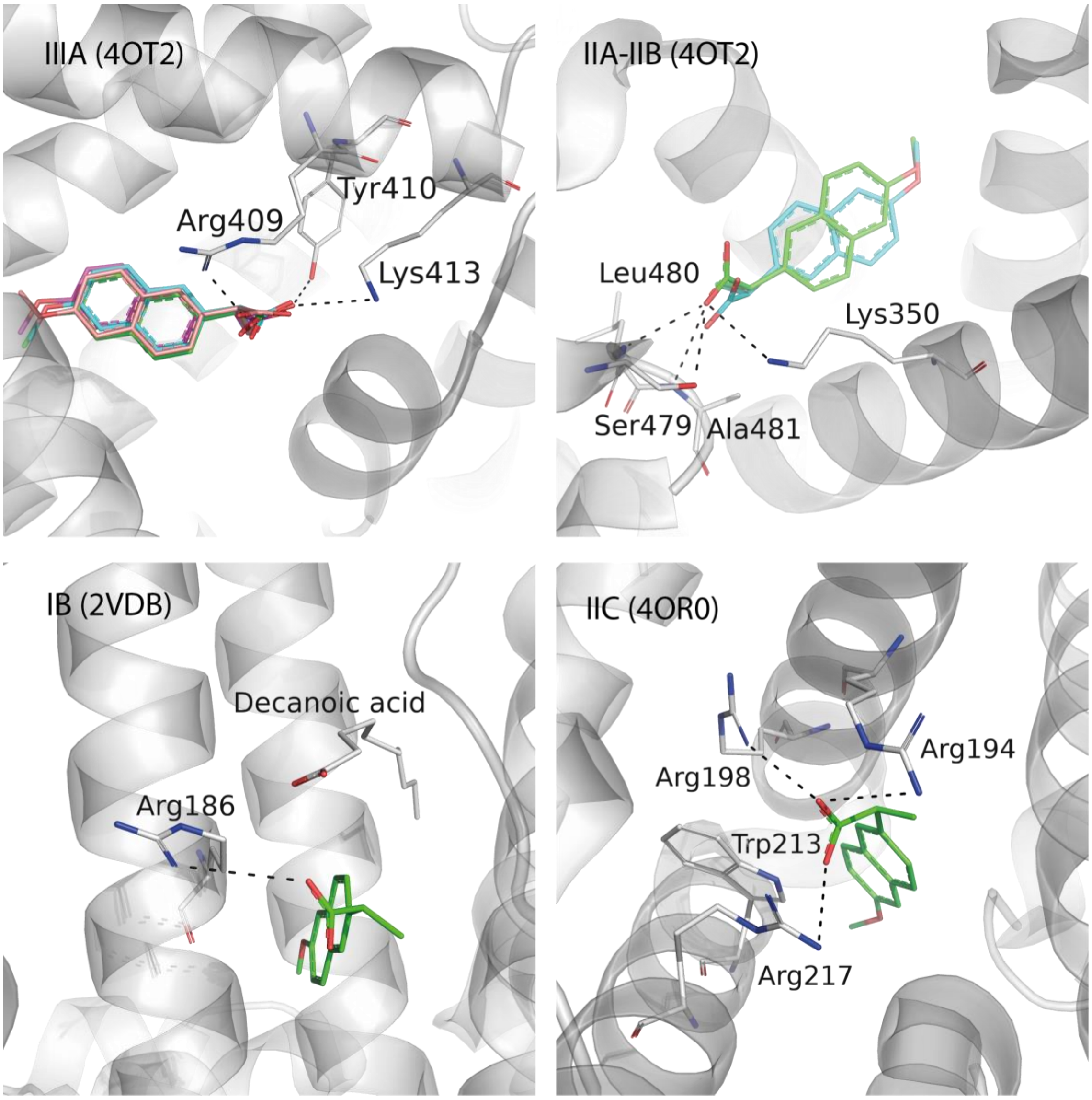

**Figure S4.** Representation of the binding mode of diflunisal at binding sites IIIA, IIA-IIB and IIA in HSA (PDB entry 2BXE). The crystallographic pose of diflunisal is shown with C atoms as colored sticks. Selected interactions with HSA residues are represented displaying the residues with C atoms as grey sticks, and interactions between residues as dashed lines. The protein backbone is displayed as grey cartoon. See Table 6 for details of binding site occupancy.

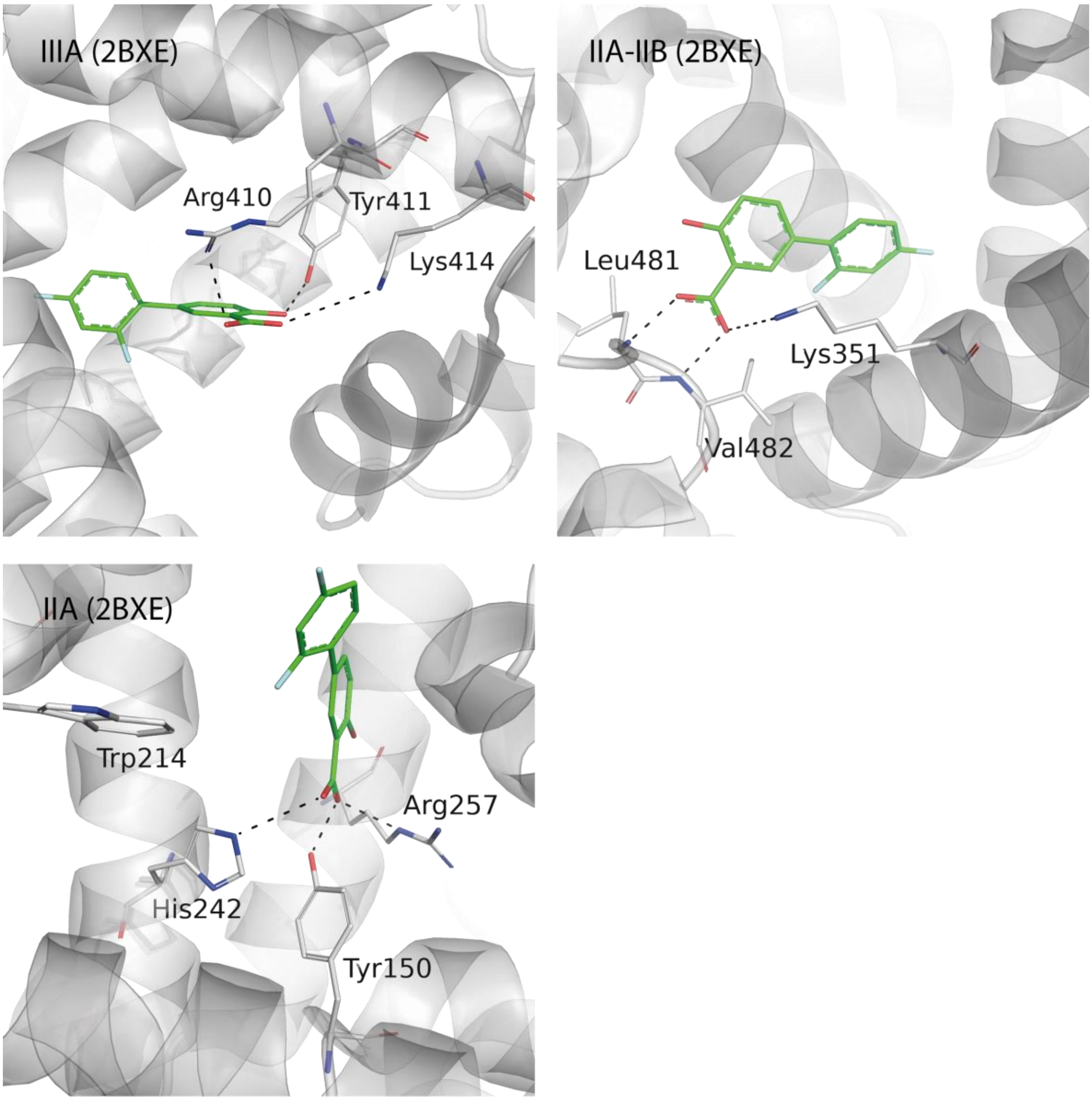

**Figure S5.** Representation of the X-ray structure 4OT2, which corresponds to HSA with diclofenac (shown with C atoms as orange sticks) bound at sites IIA and IIA-IIB. Trp214 is highlighted with C atoms as green spheres. Dicarboxylic acids and other cosolutes found in the crystallographic structures are shown as spheres.

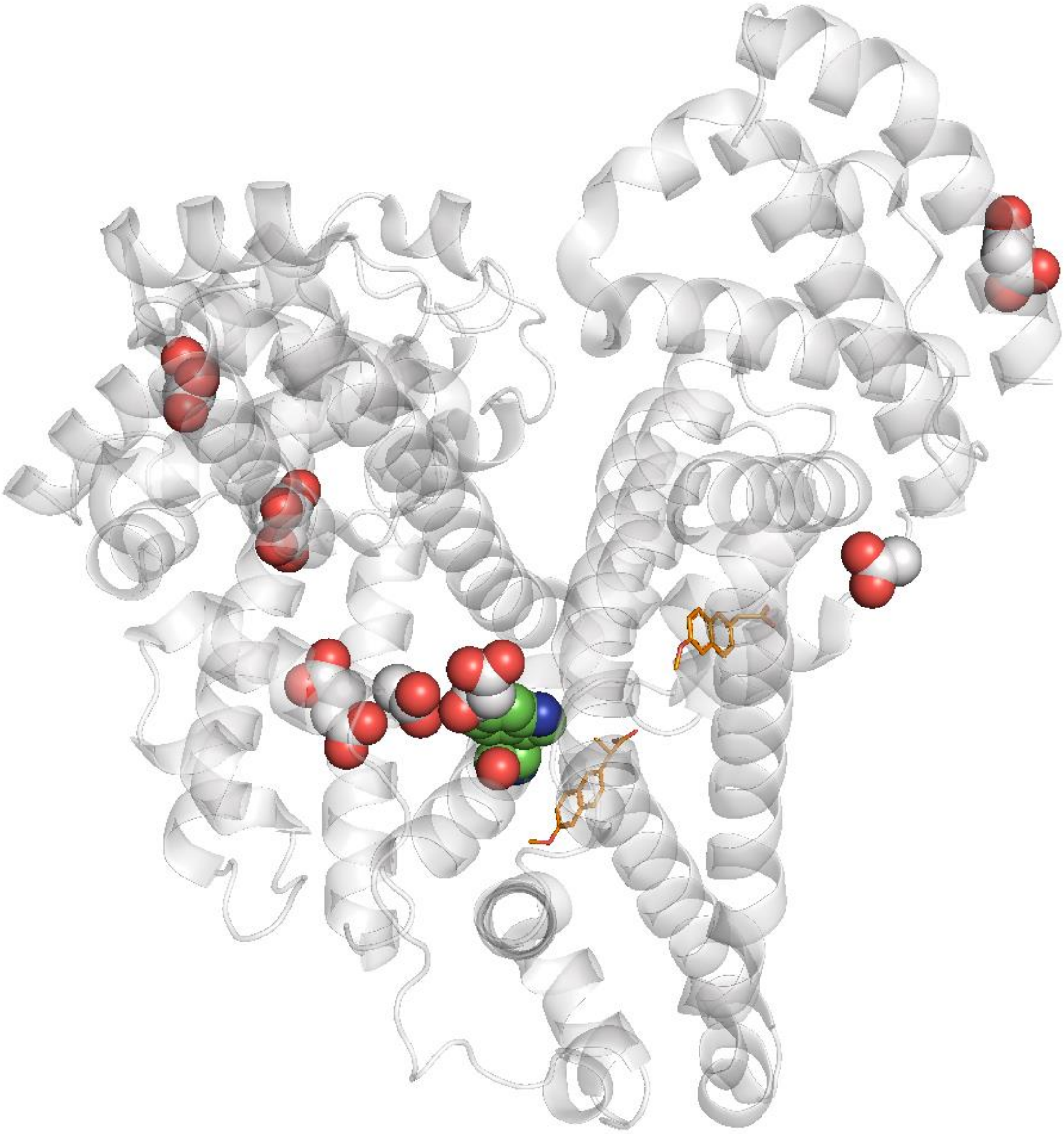

**Figure S6.** Superposition of the X-ray structures of HSA 2BXE (green ribbon) and 4Z69 (blue ribbon). The location of Trp214 in site IIC is also highlighted with C atoms as spheres. The plot reveals the structural changes triggered on several helices that shape site IIC due to the insertion of molecules of palmitic acid and pentadecanoic acid (shown with C atoms as blue spheres) in 4Z69.

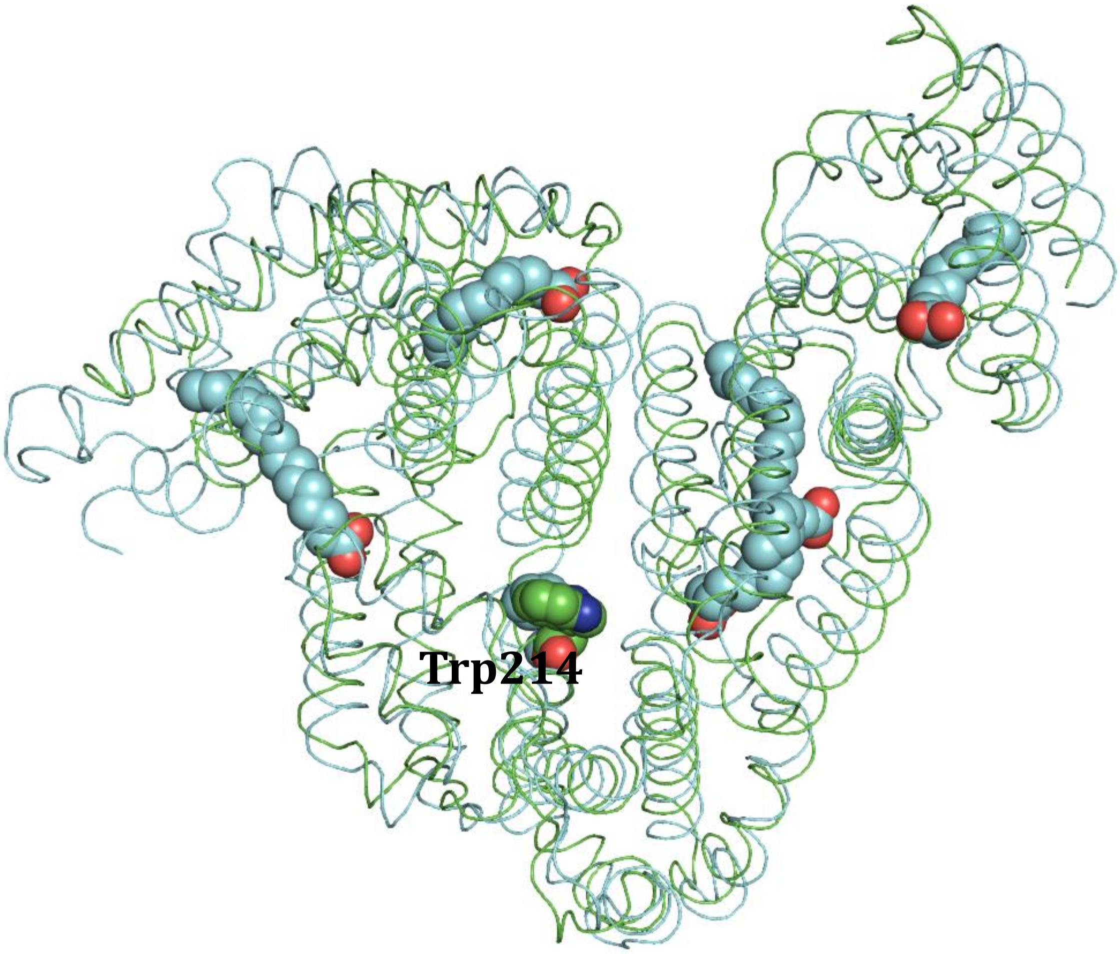

**Table S1**. Surface (Å$^2$) and volume (Å$^3$) of the compounds selected for structural analysis.

| Compound | Surface | Volume |
|---|---|---|
| Ibuprofen | 296 | 208 |
| Naproxen | 285 | 210 |
| Diflunisal | 247 | 196 |
| Diclofenac | 299 | 236 |
| Etodolac | 371 | 267 |
| 6-MNA | 260 | 194 |
| CMPF | 314 | 218 |
| Ketoprofen | 306 | 232 |
| Indomethacin | 388 | 302 |
| Suprofen | 295 | 223 |
| 3,5-diiodo salicylic acid | 171 | 174 |
| Average [a] | 306±44 | 229±33 |

[a] Determined upon exclusion of the smallest compound, 3,5-diiodo salicylic acid

**Table S2**. Comparison of sequence similarity between the serum albumin of different organisms compared to the human protein.

| Description | Uniprot code | Query Coverage | Identity | Positives |
|---|---|---|---|---|
| *E. caballus* | P35747 | 99% | 77% | 88% |
| *O. cuniculus* | P49065 | 99% | 75% | 88% |
| *B. taurus* | P02769 | 99% | 76% | 88% |
| *O. aries* | P14639 | 99% | 76% | 87% |
| *C. hircus* | B3VHM9 | 95% | 75% | 87% |

*Homo sapiens* versus *Equus caballus*

**Identities:467/608(77%), Positives:537/608(88%), Gaps:1/608(0%)**

**Query 1 MKWVTFISLLFLFSSAYSRGVFRRDAHKSEVAHRFKDLGEENFKALVLIAFAQYLQQCPF 60**
**MKWVTF+SLLFLFSSAYSRGV RRD HKSE+AHRF DLGE++FK LVL+AF+QYLQQCPF**
**Sbjct 1 MKWVTFVSLLFLFSSAYSRGVLRRDTHKSEIAHRFNDLGEKHFKGLVLVAFSQYLQQCPF 60**

**Query 61 EDHVKLVNEVTEFAKTCVADESAENCDKSLHTLFGDKLCTVATLRETYGEMADCCAKQEP 120**
**EDHVKLVNEVTEFAK C ADESAENCDKSLHTLFGDKLCTVATLR TYGE+ADCC KQEP**
**Sbjct 61 EDHVKLVNEVTEFAKKCAADESAENCDKSLHTLFGDKLCTVATLRATYGELADCCEKQEP 120**

**Query 121 ERNECFLQHKDDNPNLPRLVRPEVDVMCTAFHDNEETFLKKYLYEIARRHPYFYAPELLF 180**
**ERNECFL HKDD+PNLP+L +PE D C AF ++ + FL KYLYE+ARRHPYFY PELLF**
**Sbjct 121 ERNECFLTHKDDHPNLPKL-KPEPDAQCAAFQEDPDKFLGKYLYEVARRHPYFYGPELLF 179**

**Query 181 FAKRYKAAFTECCQAADKAACLLPKLDELRDEGKASSAKQRLKCASLQKFGERAFKAWAV 240**
**A+ YKA FTECC A DK ACL+PKLD L++ SSAK+RLKC+S Q FGERA KAW+V**
**Sbjct 180 HAEEYKADFTECCPADDKLACLIPKLDALKERILLSSAKERLKCSSFQNFGERAVKAWSV 239**

**Query 241 ARLSQRFPKAEFAEVSKLVTDLTKVHTECCHGDLLECADDRADLAKYICENQDSISSKLK 300**
**ARLSQ+FPKA+FAEVSK+VTDLTKVH ECCHGDLLECADDRADLAKYICE+QDSIS KLK**
**Sbjct 240 ARLSQKFPKADFAEVSKIVTDLTKVHKECCHGDLLECADDRADLAKYICEHQDSISGKLK 299**

**Query 301 ECCEKPLLEKSHCIAEVENDEMPADLPSLAADFVESKDVCKNYAEAKDVFLGMFLYEYAR 360**
**CC+KPLL+KSHCIAEV+ D++P+DLP+LAADF E K++CK+Y +AKDVFLG FLYEY+R**
**Sbjct 300 ACCDKPLLQKSHCIAEVKEDDLPSDLPALAADFAEDKEICKHYKDAKDVFLGTFLYEYSR 359**

**Query 361 RHPDYSVVLLLRLAKTYETTLEKCCAAADPHECYAKVFDEFKPLVEEPQNLIKQNCELFE 420**
**RHPDYSV LLLR+AKTYE TLEKCCA ADP CY VFD+F PLVEEP++L+K+NC+LFE**
**Sbjct 360 RHPDYSVSLLLRIAKTYEATLEKCCAEADPPACYRTVFDQFTPLVEEPKSLVKKNCDLFE 419**

**Query 421 QLGEYKFQNALLVRYTKKVPQVSTPTLVEVSRNLGKVGSKCCKHPEAKRMPCAEDYLSVV 480**
**++GEY FQNAL+VRYTKK PQVSTPTLVE+ R LGKVGS+CCK PE++R+PC+E++L++**
**Sbjct 420 EVGEYDFQNALIVRYTKKAPQVSTPTLVEIGRTLGKVGSRCCKLPESERLPCSENHLALA 479**

**Query 481 LNQLCVLHEKTPVSDRVTKCCTESLVNRRPCFSALEVDETYVPKEFNAETFTFHADICTL 540**
**LN+LCVLHEKTPVS+++TKCCT+SL RRPCFSALE+DE YVPKEF AETFTFHADICTL**
**Sbjct 480 LNRLCVLHEKTPVSEKITKCCTDSLAERRPCFSALELDEGYVPKEFKAETFTFHADICTL 539**

**Query 541 SEKERQIKKQTALVELVKHKPKATKEQLKAVMDDFAAFVEKCCKADDKETCFAEEGKKLV 600**
**E E+QIKKQ+AL ELVKHKPKATKEQLK V+ +F+AFV KCC +DKE CFAEEG KLV**
**Sbjct 540 PEDEKQIKKQSALAELVKHKPKATKEQLKTVLGNFSAFVAKCCGREDKEACFAEEGPKLV 599**

**Query 601 AASQAALG 608**
**A+SQ AL**
**Sbjct 600 ASSQLALA 607**

*Homo sapiens* versus *Bos taurus*

**Identities:465/608(76%), Positives:536/608(88%), Gaps:1/608(0%)**

```
Query  1    MKWVTFISLLFLFSSAYSRGVFRRDAHKSEVAHRFKDLGEENFKALVLIAFAQYLQQCPF
60
            MKWVTFISLL LFSSAYSRGVFRRD HKSE+AHRFKDLGEE+FK LVLIAF+QYLQQCPF
Sbjct  1    MKWVTFISLLLLFSSAYSRGVFRRDTHKSEIAHRFKDLGEEHFKGLVLIAFSQYLQQCPF  60

Query  61   EDHVKLVNEVTEFAKTCVADESAENCDKSLHTLFGDKLCTVATLRETYGEMADCCAKQEP
120
            ++HVKLVNE+TEFAKTCVADES   C+KSLHTLFGD+LC VA+LRETYG+MADCC KQEP
Sbjct  61   DEHVKLVNELTEFAKTCVADESHAGCEKSLHTLFGDELCKVASLRETYGDMADCCEKQEP
120

Query  121  ERNECFLQHKDDNPNLPRLVRPEVDVMCTAFHDNEETFLKKYLYEIARRHPYFYAPELLF
180
            ERNECFL HKDD+P+LP+L +P+ + +C  F  +E+ F  KYLYEIARRHPYFYAPELL+
Sbjct  121  ERNECFLSHKDDSPDLPKL-KPDPNTLCDEFKADEKKFWGKYLYEIARRHPYFYAPELLY
179

Query  181
FAKRYKAAFTECCQAADKAACLLPKLDELRDEGKASSAKQRLKCASLQKFGERAFKAWAV  240
            +A +Y   F ECCQA DK ACLLPK++ +R++  ASSA+QRL+CAS+QKFGERA KAW+V
Sbjct  180  YANKYNGVFQECCQAEDKGACLLPKIETMREKVLASSARQRLRCASIQKFGERALKAWSV
239

Query  241  ARLSQRFPKAEFAEVSKLVTDLTKVHTECCHGDLLECADDRADLAKYICENQDSISSKLK
300
            ARLSQ+FPKAEF EV+KLVTDLTKVH ECCHGDLLECADDRADLAKYIC+NQD+ISSKLK
Sbjct  240  ARLSQKFPKAEFVEVTKLVTDLTKVHKECCHGDLLECADDRADLAKYICDNQDTISSKLK
299

Query  301  ECCEKPLLEKSHCIAEVENDEMPADLPSLAADFVESKDVCKNYAEAKDVFLGMFLYEYAR
360
            ECC+KPLLEKSHCIAEVE D +P +LP L ADF E KDVCKNY EAKD FLG FLYEY+R
Sbjct  300  ECCDKPLLEKSHCIAEVEKDAIPENLPPLTADFAEDKDVCKNYQEAKDAFLGSFLYEYSR
359

Query  361  RHPDYSVVLLLRLAKTYETTLEKCCAAADPHECYAKVFDEFKPLVEEPQNLIKQNCELFE
420
            RHP+Y+V +LLRLAK YE TLE+CCA  DPH CY+ VFD+ K LV+EPQNLIKQNC+ FE
Sbjct  360  RHPEYAVSVLLRLAKEYEATLEECCAKDDPHACYSTVFDKLKHLVDEPQNLIKQNCDQFE
419

Query  421  QLGEYKFQNALLVRYTKKVPQVSTPTLVEVSRNLGKVGSKCCKHPEAKRMPCAEDYLSVV
480
            +LGEY FQNAL+VRYT+KVPQVSTPTLVEVSR+LGKVG++CC  PE++RMPC EDYLS++
Sbjct  420  KLGEYGFQNALIVRYTRKVPQVSTPTLVEVSRSLGKVGTRCCTKPESERMPCTEDYLSLI
479

Query  481  LNQLCVLHEKTPVSDRVTKCCTESLVNRRPCFSALEVDETYVPKEFNAETFTFHADICTL
540
            LN+LCVLHEKTPVS++VTKCCTESLVNRRPCFSAL  DETYVPK F+ + FTFHADICTL
Sbjct  480  LNRLCVLHEKTPVSEKVTKCCTESLVNRRPCFSALTPDETYVPKAFDEKLFTFHADICTL
539

Query  541  SEKERQIKKQTALVELVKHKPKATKEQLKAVMDDFAAFVEKCCKADDKETCFAEEGKKLV
600
             + E+QIKKQTALVEL+KHKPKAT+EQLK VM++F AFV+KCC ADDKE CFA EG KLV
Sbjct  540  PDTEKQIKKQTALVELLKHKPKATEEQLKTVMENFVAFVDKCCAADDKEACFAVEGPKLV
599

Query  601  AASQAALG  608
             ++Q AL
```

**Sbjct  600  VSTQTALA  607**

**Homo sapiens versus Oryctolagus cuniculus**

**Identities:458/608(75%), Positives:536/608(88%), Gaps:0/608(0%)**

**Query 1 MKWVTFISLLFLFSSAYSRGVFRRDAHKSEVAHRFKDLGEENFKALVLIAFAQYLQQCPF 60**
**MKWVTFISLLFLFSSAYSRGVFRR+AHKSE+AHRF D+GEE+F LVLI F+QYLQ+CP+**
**Sbjct 1 MKWVTFISLLFLFSSAYSRGVFRREAHKSEIAHRFNDVGEEHFIGLVLITFSQYLQKCPY 60**

**Query 61 EDHVKLVNEVTEFAKTCVADESAENCDKSLHTLFGDKLCTVATLRETYGEMADCCAKQEP 120**
**E+H KLV EVT+ AK CVADESA NCDKSLH +FGDK+C + +LR+TYG++ADCC K+EP**
**Sbjct 61 EEHAKLVKEVTDLAKACVADESAANCDKSLHDIFGDKICALPSLRDTYGDVADCCEKKEP 120**

**Query 121 ERNECFLQHKDDNPNLPRLVRPEVDVMCTAFHDNEETFLKKYLYEIARRHPYFYAPELLF 180**
**ERNECFL HKDD P+LP RPE DV+C AFHD+E+ F YLYE+ARRHPYFYAPELL+**
**Sbjct 121 ERNECFLHHKDDKPDLPPFARPEADVLCKAFHDDEKAFFGHYLYEVARRHPYFYAPELLY 180**

**Query 181 FAKRYKAAFTECCQAADKAACLLPKLDELRDEGKASSAKQRLKCASLQKFGERAFKAWAV 240**
**+A++YKA TECC+AADK ACL PKLD L + S+A++RL+CAS+QKFG+RA+KAWA+**
**Sbjct 181 YAQKYKAILTECCEAADKGACLTPKLDALEGKSLISAAQERLRCASIQKFGDRAYKAWAL 240**

**Query 241 ARLSQRFPKAEFAEVSKLVTDLTKVHTECCHGDLLECADDRADLAKYICENQDSISSKLK 300**
**RLSQRFPKA+F ++SK+VTDLTKVH ECCHGDLLECADDRADLAKY+CE+Q++ISS LK**
**Sbjct 241 VRLSQRFPKADFTDISKIVTDLTKVHKECCHGDLLECADDRADLAKYMCEHQETISSHLK 300**

**Query 301 ECCEKPLLEKSHCIAEVENDEMPADLPSLAADFVESKDVCKNYAEAKDVFLGMFLYEYAR 360**
**ECC+KP+LEK+HCI + NDE PA LP++A +FVE KDVCKNY EAKD+FLG FLYEY+R**
**Sbjct 301 ECCDKPILEKAHCIYGLHNDETPAGLPAVAEEFVEDKDVCKNYEEAKDLFLGKFLYEYSR 360**

**Query 361 RHPDYSVVLLLRLAKTYETTLEKCCAAADPHECYAKVFDEFKPLVEEPQNLIKQNCELFE 420**
**RHPDYSVVLLLRL K YE TL+KCCA DPH CYAKV DEF+PLV+EP+NL+KQNCEL+E**
**Sbjct 361 RHPDYSVVLLLRLGKAYEATLKKCCATDDPHACYAKVLDEFQPLVDEPKNLVKQNCELYE 420**

**Query 421 QLGEYKFQNALLVRYTKKVPQVSTPTLVEVSRNLGKVGSKCCKHPEAKRMPCAEDYLSVV 480**
**QLG+Y FQNALLVRYTKKVPQVSTPTLVE+SR+LGKVGSKCCKHPEA+R+PC EDYLSVV**
**Sbjct 421 QLGDYNFQNALLVRYTKKVPQVSTPTLVEISRSLGKVGSKCCKHPEAERLPCVEDYLSVV 480**

**Query 481 LNQLCVLHEKTPVSDRVTKCCTESLVNRRPCFSALEVDETYVPKEFNAETFTFHADICTL 540**
**LN+LCVLHEKTPVS++VTKCC+ESLV+RRPCFSAL DETYVPKEFNAETFTFHADICTL**
**Sbjct 481 LNRLCVLHEKTPVSEKVTKCCSESLVDRRPCFSALGPDETYVPKEFNAETFTFHADICTL 540**

**Query 541 SEKERQIKKQTALVELVKHKPKATKEQLKAVMDDFAAFVEKCCKADDKETCFAEEGKKLV 600**
**E ER+IKKQTALVELVKHKP AT +QLK V+ +F A ++KCC A+DKE CFA EG KLV**
**Sbjct 541 PETERKIKKQTALVELVKHKPHATNDQLKTVVGEFTALLDKCCSAEDKEACFAVEGPKLV 600**

**Query 601 AASQAALG 608**
**+S+A LG**

**Sbjct 601 ESSKATLG 608**

***Homo sapiens* versus *Ovis aries***

**Identities:460/608(76%), Positives:533/608(87%), Gaps:1/608(0%)**

**Query 1 MKWVTFISLLFLFSSAYSRGVFRRDAHKSEVAHRFKDLGEENFKALVLIAFAQYLQQCPF 60**
**MKWVTFISLL LFSSAYSRGVFRRD HKSE+AHRF DLGEENF+ LVLIAF+QYLQQCPF**
**Sbjct 1 MKWVTFISLLLLFSSAYSRGVFRRDTHKSEIAHRFNDLGEENFQGLVLIAFSQYLQQCPF 60**

**Query 61 EDHVKLVNEVTEFAKTCVADESAENCDKSLHTLFGDKLCTVATLRETYGEMADCCAKQEP 120**
**++HVKLV E+TEFAKTCVADES CDKSLHTLFGD+LC VATLRETYG+MADCC KQEP**
**Sbjct 61 DEHVKLVKELTEFAKTCVADESHAGCDKSLHTLFGDELCKVATLRETYGDMADCCEKQEP 120**

**Query 121 ERNECFLQHKDDNPNLPRLVRPEVDVMCTAFHDNEETFLKKYLYEIARRHPYFYAPELLF 180**
**ERNECFL HKDD+P+LP+L +PE D +C F +E+ F KYLYE+ARRHPYFYAPELL+**
**Sbjct 121 ERNECFLNHKDDSPDLPKL-KPEPDTLCAEFKADEKKFWGKYLYEVARRHPYFYAPELLY 179**

**Query 181 FAKRYKAAFTECCQAADKAACLLPKLDELRDEGKASSAKQRLKCASLQKFGERAFKAWAV 240**
**+A +Y F ECCQA DK ACLLPK+D +R++ ASSA+QRL+CAS+QKFGERA KAW+V**
**Sbjct 180 YANKYNGVFQECCQAEDKGACLLPKIDAMREKVLASSARQRLRCASIQKFGERALKAWSV 239**

**Query 241 ARLSQRFPKAEFAEVSKLVTDLTKVHTECCHGDLLECADDRADLAKYICENQDSISSKLK 300**
**ARLSQ+FPKA+F +V+K+VTDLTKVH ECCHGDLLECADDRADLAKYIC++QD++SSKLK**
**Sbjct 240 ARLSQKFPKADFTDVTKIVTDLTKVHKECCHGDLLECADDRADLAKYICDHQDALSSKLK 299**

**Query 301 ECCEKPLLEKSHCIAEVENDEMPADLPSLAADFVESKDVCKNYAEAKDVFLGMFLYEYAR 360**
**ECC+KP+LEKSHCIAEV+ D +P +LP L ADF E K+VCKNY EAKDVFLG FLYEY+R**
**Sbjct 300 ECCDKPVLEKSHCIAEVDKDAVPENLPPLTADFAEDKEVCKNYQEAKDVFLGSFLYEYSR 359**

**Query 361 RHPDYSVVLLLRLAKTYETTLEKCCAAADPHECYAKVFDEFKPLVEEPQNLIKQNCELFE 420**
**RHP+Y+V +LLRLAK YE TLE CCA DPH CYA VFD+ K LV+EPQNLIK+NCELFE**
**Sbjct 360 RHPEYAVSVLLRLAKEYEATLEDCCAKEDPHACYATVFDKLKHLVDEPQNLIKKNCELFE 419**

**Query 421 QLGEYKFQNALLVRYTKKVPQVSTPTLVEVSRNLGKVGSKCCKHPEAKRMPCAEDYLSVV 480**
**+ GEY FQNAL+VRYT+K PQVSTPTLVE+SR+LGKVG+KCC PE++RMPC EDYLS++**
**Sbjct 420 KHGEYGFQNALIVRYTRKAPQVSTPTLVEISRSLGKVGTKCCAKPESERMPCTEDYLSLI 479**

**Query 481 LNQLCVLHEKTPVSDRVTKCCTESLVNRRPCFSALEVDETYVPKEFNAETFTFHADICTL 540**
**LN+LCVLHEKTPVS++VTKCCTESLVNRRPCFS L +DETYVPK F+ + FTFHADICTL**
**Sbjct 480 LNRLCVLHEKTPVSEKVTKCCTESLVNRRPCFSDLTLDETYVPKPFDEKFFTFHADICTL 539**

**Query 541 SEKERQIKKQTALVELVKHKPKATKEQLKAVMDDFAAFVEKCCKADDKETCFAEEGKKLV 600**
**+ E+QIKKQTALVEL+KHKPKAT EQLK VM++F AFV+KCC ADDKE CF EG KLV**
**Sbjct 540 PDTEKQIKKQTALVELLKHKPKATDEQLKTVMENFVAFVDKCCAADDKEGCFVLEGPKLV 599**

**Query 601 AASQAALG 608**
**A++QAAL**

Sbjct  600  ASTQAALA  607

***Homo sapiens*** **versus** ***Capra hircus***

**Identities:436/584(75%), Positives:512/584(87%), Gaps:1/584(0%)**

```
Query  25   DAHKSEVAHRFKDLGEENFKALVLIAFAQYLQQCPFEDHVKLVNEVTEFAKTCVADESAE
84
        D HKSE+AHRF DLGEENF+ LVLIAF+QYLQQCPF++HVKLV E+TEFAKTCVADES
Sbjct  1    DTHKSEIAHRFNDLGEENFQGLVLIAFSQYLQQCPFDEHVKLVKELTEFAKTCVADESHA  60

Query  85   NCDKSLHTLFGDKLCTVATLRETYGEMADCCAKQEPERNECFLQHKDDNPNLPRLVRPEV
144
         CDKSLHTLFGD+LC VATLRETYG+MADCC KQEPERNECFL+HKDD+P+LP+L +PE
Sbjct  61   GCDKSLHTLFGDELCKVATLRETYGDMADCCEKQEPERNECFLKHKDDSPDLPKL-KPEP
119

Query  145  DVMCTAFHDNEETFLKKYLYEIARRHPYFYAPELLFFAKRYKAAFTECCQAADKAACLLP
204
        D +C  F  +E+ F  KYLYE+ARRHPYFYAPELL++A +Y   F ECCQA DK ACLLP
Sbjct  120  DTLCAEFKADEKKFWGKYLYEVARRHPYFYAPELLYYANKYNGVFQECCQAEDKGACLLP
179

Query  205
KLDELRDEGKASSAKQRLKCASLQKFGERAFKAWAVARLSQRFPKAEFAEVSKLVTDLTK  264
        K++ +R++  ASSA+QRL+CAS+QKFGERA KAW+VARLSQ+FPKA+F +V+K+VTDLTK
Sbjct  180  KIETMREKVLASSARQRLRCASIQKFGERALKAWSVARLSQKFPKADFTDVTKIVTDLTK
239

Query  265  VHTECCHGDLLECADDRADLAKYICENQDSISSKLKECCEKPLLEKSHCIAEVENDEMPA
324
        VH ECCHGDLLECADDRADLAKYIC++QD++SSKLKECC+KP+LEKSHCIAE++ D +P
Sbjct  240  VHKECCHGDLLECADDRADLAKYICDHQDTLSSKLKECCDKPVLEKSHCIAEIDKDAVPE
299

Query  325  DLPSLAADFVESKDVCKNYAEAKDVFLGMFLYEYARRHPDYSVVLLLRLAKTYETTLEKC
384
        +LP L ADF E K+VCKNY EAKDVFLG FLYEY+RRHP+Y+V +LLRLAK YE TLE C
Sbjct  300  NLPPLTADFAEDKEVCKNYQEAKDVFLGSFLYEYSRRHPEYAVSVLLRLAKEYEATLEDC
359

Query  385  CAAADPHECYAKVFDEFKPLVEEPQNLIKQNCELFEQLGEYKFQNALLVRYTKKVPQVST
444
        CA  DPH CYA VFD+ K LV+EPQNLIK+NCELFE+ GEY FQNAL+VRYT+K PQVST
Sbjct  360  CAKEDPHACYATVFDKLKHLVDEPQNLIKKNCELFEKHGEYGFQNALIVRYTRKAPQVST
419

Query  445  PTLVEVSRNLGKVGSKCCKHPEAKRMPCAEDYLSVVLNQLCVLHEKTPVSDRVTKCCTES
504
        PTLVE+SR+LGKVG+KCC  PE++RMPC EDYLS++LN+LCVLHEKTPVS++VTKCCTES
Sbjct  420  PTLVEISRSLGKVGTKCCAKPESERMPCTEDYLSLILNRLCVLHEKTPVSEKVTKCCTES
479

Query  505  LVNRRPCFSALEVDETYVPKEFNAETFTFHADICTLSEKERQIKKQTALVELVKHKPKAT
564
        LVNRRPCFS L +DETYVPK F+ E+FTFHADICTL + E+QIKKQTALVEL+KHKPKAT
Sbjct  480  LVNRRPCFSDLTLDETYVPKPFDGESFTFHADICTLPDTEKQIKKQTALVELLKHKPKAT
539

Query  565  KEQLKAVMDDFAAFVEKCCKADDKETCFAEEGKKLVAASQAALG  608
         EQLK VM++F AFV+KCC ADDKE CF  EG KLVA++QAAL
Sbjct  540  DEQLKTVMENFVAFVDKCCAADDKEGCFLLEGPKLVASTQAALA  583
```
